# Exploring food system resilience to the global polycrisis in six Asian countries

**DOI:** 10.3389/fnut.2024.1347186

**Published:** 2024-04-16

**Authors:** Caroline Favas, Chiara Cresta, Elizabeth Whelan, Kristie Smith, Mari S. Manger, Damith Chandrasenage, Anusara Singhkumarwong, Jintana Kawasaki, Susana Moreno, Sophie Goudet

**Affiliations:** ^1^Dikoda Ltd., Nutrition Research, London, United Kingdom; ^2^World Food Programme Regional Bureau for Asia and the Pacific, Bangkok, Thailand

**Keywords:** food system, polycrisis, food system resilience, NCD-protect, NCD-risk

## Abstract

The world is currently in the midst of a global food crisis brought about and exacerbated by a series of mutually reinforcing shocks to food systems This study investigated the resilience of food systems in six Asian countries (Bangladesh, Kyrgyz Republic, Lao PDR, Pakistan, Philippines, and Sri Lanka) amidst the global ‘polycrisis’ caused by COVID-19, geopolitical conflicts, and climate change. Trend analyses were performed for 19 indicators sourced from global databases and World Food Programme national data, representing the four domains of food system resilience: exposure to shocks; resilience capacities and agro- and food diversity, resilience responses and strategies; and long-term resilience outcomes. The analysis revealed that all six countries experienced the effects of the ‘polycrisis’, leading to diverse impacts on exchange rates, with Sri Lanka, Pakistan, and Lao PDR facing significant currency depreciation. While most countries increased crop production and decreased food imports during the crisis, government economic support during the pandemic varied widely. Resilience outcomes, including national food price inflation and the proportion of populations facing food insecurity, witnessed upward variations. Overall, countries with higher resilience capacities at the start of the ‘polycrisis’ showed less severe long-term resilience outcomes. Our findings highlight the varied challenges and resilience capacities across each country, influenced by a complex interplay of economic, political, agricultural, and food affordability factors crucial for determining long-term resilience in their food systems. Recommendations for future research include focusing on resilience assessment in food systems, integrating climate change adaptation measures, and developing early intervention strategies.

## 1 Introduction

The unfolding global food crisis, triggered by a sequence of interconnected shocks including the COVID-19 pandemic, geopolitical conflicts, and climate-induced extreme weather events, has severely disrupted food systems worldwide (1). The war in Ukraine exacerbated the strain on global supply chains, leading to critical shortages in supply stocks. Simultaneously, geopolitical shifts influenced by the conflict disrupted worldwide energy provisions, resulting in increased costs for food processing, transportation, and commodity prices (2). Climate change-related extreme weather events further compounded these challenges, diminishing global food production and wreaking havoc on critical infrastructure (3, 4). The simultaneous occurrence of these events, defined as a ‘polycrisis’ (5), has spurred significant spikes in food price inflation and heightened food insecurity globally (6), with the Asia-Pacific region in particular experiencing swift and severe impacts (7, 8).

The region, already burdened with the world’s highest rates of all forms of malnutrition, has seen millions more people becoming acutely food-insecure in the past year (9). Supply chain disruptions, protectionist trade measures, and climate-induced events have contributed further to the region’s crisis (10, 11) and necessitates immediate attention and innovative solutions to address the unprecedented challenges.

To mitigate the crisis and enhance long-term food security against climate change and biodiversity loss, the Asian Development Bank has announced plans to provide at least $14 billion over 2022–2025 to bolster food systems in the region (12). To ensure investments are strategically directed, it is crucial to explore how food systems have reacted to shocks, and how resilience can be enhanced to ensure long-term food security in the face of future crises.

The objective of this study was to explore the resilience of food systems to the ongoing global polycrisis in six countries in the Asia region (Bangladesh, Kyrgyz Republic, Lao PDR, Pakistan, Philippines, and Sri Lanka).

## 2 Methods

### 2.1 Conceptual framework

This study employed a conceptual framework adapted from the model developed by Fanzo et al. (13) to assess the resilience of food systems amid ongoing crises, utilizing a holistic, national-level approach, building on work by many others (14–17). As defined by Fanzo et al. 2021, food system resilience is “the ability of different individual and institutional food system actors to maintain, protect, or quickly recover the key functions of that system despite the impacts of disturbances.”

This model of food system resilience encompasses four indicator domains: (1) exposure to shocks; (2) resilience capacities and agro-and food diversity; (3) resilience responses or strategies; and (4) long-term outcomes (Table 1).

**Table 1:** Description of food systems resilience components (13).

Indicator domain for food system resilience	Description
Exposure to shocks	Assessment of food system resilience begins with assessing and documenting the adverse effects that affect those systems.
Resilience Capacities	Features that are expected to make a system or its actors more resilient. Characteristics include redundancy, diversity, flexibility, connectivity, anticipation, self-efficacy, or access to insurance or formal credit. Potential indicators of resilience capacities include food system actors’ adaptive capacities (e. g., connectivity, social capital), social cohesion, or measures of value chain flexibility.
Agro- and Food Diversity	Agrobiodiversity and food diversity play important roles in building resilience in crop, livestock, forest, fishery, and aquaculture production systems. Moreover, agrobiodiversity secures the resilience capabilities of food systems for future generations and yet-unknown shocks. Food diversity underpins healthy diets with most food based dietary guidelines recommending a diet that is high in food group diversity.
Resilience Responses and Strategies	Not all responses adopted by individuals, communities, or societies to anticipate or mitigate/buffer the impact of shocks and stressors result in positive outcomes, either in the short- or longer-term. Understanding and measuring resilience also requires documenting and tracking these responses as an attempt to strengthen the ability of households and society to choose what they perceive at that time as the “right” response(s), rather than being forced by circumstances or lack of information to pick options which might be detrimental overall.
Long-term outcomes	The indicators used to measure long-term outcomes should reflect these improvements in human well-being. In the context of food systems many of those improvements will revolve around food security and nutrition. Tracking the stability of the different pillars of food security (food availability, access, and utilization) could therefore be an important element in measuring food system resilience, but it could include other indicators such as the stability in the livelihoods of these involved in food system activities. The emphasis, however, should be on measuring (in)stability over time rather than absolute values.

### 2.2 Indicator selection and data

Indicators for each food system domain were adapted from Schneider et al. (18) and customized for the specific context of the six selected countries (Table 2). The inclusion criteria were as follows:

- Relevance: Indicators that could clearly be linked to the fundamental components of the food system dashboard framework (19): External Factors (drivers), Food Environments, Food Supply Chains, Individual Factors, Outcomes.
- Availability of data: Indicators for which data was available for both the “pre-crisis” period (2010–2019) and the “ongoing crisis” period (2020–2023), and if possible, information was available for all six countries.

**Table 2:** Food system resilience indicators.

Indicator domain	Sub domain	Indicator	Data source
Exposure to shocks	Economic stability	Exchange rate	FAOSTAT
	Natural disaster	Ratio of affected people to the total population	EM-DAT
	COVID-19	COVID-19 Stringency Index	OxCGRT
Resilience capacities and agro-food diversity	Food produced domestically	Crop production index (2014–2016 = 100)	World Bank
		Fertilizer consumption	World Bank
		Livestock production index (2014–2016 = 100)	World Bank
	Imported food	Food import (volume) NCD-protect Food import (volume) NCD-risk food groups	UN Comtrade
	Countries’ infrastructure level	Mobile cellular subscription	World Bank
	Social capital	Social capital index	Legatum Institute / FSCI
	COVID-19	COVID-19 Economic Support Index	OxCGRT
Resilience responses and strategies	Coping strategy	Reduced Coping Strategy Index (rCSI)*	WFP
		Livelihood Coping Strategy (LCS)**	WFP
Longer-term resilience outcomes	Food price volatility	Food price inflation	FAOSTAT
		Food Price Anomalies (IFPA), by type of product (Rice)	FAOSTAT
		Food Price Anomalies (IFPA), by type of product (Wheat)	FAOSTAT
	Food supply variability	Food supply variability	FAOSTAT
	Food security	% population experiencing moderate or severe food insecurity	FAOSTAT
		% population who cannot afford a healthy diet	FAOSTAT
		Food consumption score (FCS)***	WFP

*Reduced coping strategy index: this indicator measures the frequency and severity of household behaviors when faced with shortages of food or financial resources to buy food. A higher score indicates more frequent and/or extreme negative coping strategies. Based on the score it is categorized in: No/low coping strategy (rCSI = 0–3); Medium coping strategy (rCSI = 4–18); High coping strategy (rCSI > 18). **Livelihood coping strategy: This is an indicator used to understand households’ (HH) medium and longer-term coping capacity in response to lack of food or lack of money to buy food and their ability to overcome challenges in the future. Strategies can be classified into four severity groups: HH not adopting coping strategies; HH adopting stress coping strategies; HH adopting crisis coping strategies; HH adopting emergencies coping strategies. ***Food consumption score: This indicator is a composite score based on households’ dietary diversity, food consumption frequency, and relative nutritional value of different food groups. It is a commonly used WFP indicator and was included despite data not being available for all six countries. Based on the score it is categorized in: Poor (FCS≤28); Borderline (FCS = 28.001–42); Acceptable (FCS > 42).

The data used in this study were partially sourced from various global, publicly available data sources, including the World Bank’s Health Nutrition and Population Statistics, Food and Agriculture Organization Corporate Statistical Database (FAOSTAT), Oxford Covid-19 Government Response Tracker (OxCGRT), EM-DAT The International Disaster Database, The Legatum Prosperity Index, and The United Nations Commodity Trade Statistics Database (UN Comtrade). For three indicators, the reduced Coping Strategy Index (rCSI), the Livelihood Coping Strategy-Food Security (LCS-FS), and the Food Consumption Score (FCS), publicly and non-publicly available data were provided by the World Food Program (WFP) Regional Bangkok Bureau. Through engagement with WFP country offices in the six countries, additional data sources were also identified. In cases where data for a specific indicator were unavailable, the feasibility of using one or multiple proxies was evaluated.

Data extracted from the selected global data sources, along with definitions are included in a Supplementary Table.

### 2.3 Categorizing food import data

To analyze food import from a nutritional perspective, data from UN Comtrade were reclassified based on the 28 out of 29 food groups outlined in the Global Diet Quality Project’s Diet Quality Questionnaire (DQQ) guidelines (20). The 29th DQQ food group-fast foods - was excluded as it is not considered a food consumed for purchase at home and was therefore not available in the UN Comtrade import databases.

Following the classification of food import into DQQ food groups, these groups were further categorized into three groups based on the Global Dietary Recommendations (GDR) Score. The GDR Score is a measure based on standard, evidence-based guidelines related to foods known to either contribute to or protect from noncommunicable diseases (NCDs) (21). The three GDR food categories established for this study were: “NCD protect” foods, serving to protect against NCDs, “NCD risk” foods, known to pose a risk for NCDs, and “other foods” group, including food items classified as neither NCD-risk nor NCD-protect. The detailed classification of food groups into these three GDR food categories is described in Appendix A.

### 2.4 Statistical analysis

To assess the impact of the shocks resulting from the polycrisis on selected resilience indicators, trend analyses were performed using data from 2010, when available, to the most recent available year of the ongoing crisis period. Microsoft Power BI’s time series prediction method, known as exponential smoothing, was applied to generate forecasts and 95% confidence intervals starting from 2019. A comparison was made for each resilience indicator between the actual trend direction and the desired direction. An overview of the evolution of indicators of “resilience capacities and agro- and food-diversity,” “resilience responses/strategies” and “longer-term resilience outcomes” domains from pre-crisis to crisis period in the six targeted countries is presented in Appendix A.

To further investigate changes in household food security between the “pre-crisis” and “crisis” periods, we calculated the relative percentage change in FCS from 2019. The results are presented in the form of boxplots in Section 3.4.2. “Pre-crisis” and “crisis” period data for FCS were only available for three of the six targeted countries—Bangladesh, the Kyrgyz Republic, and Pakistan.

To simplistically capture the explanatory power of the model, we examined whether countries with higher resilience capacities exhibit more favorable long-term outcomes than those with lower resilience capacities. For this purpose, values for all indicators within the domains *resilience capacities and agro-food diversity* and *long-term resilience outcomes* were assigned a rank from 1 to 6, with 6 being the most desirable score, for example having the highest crop production or the lowest food price inflation. The rankings for resilience capacities and agro-food diversity were based on values from the pre-crisis year 2019, while those for long-term resilience outcomes were based on values from the most recent available “crisis period” data. The Economic Support Index indicator, serving as a proxy for resilience capacities, was excluded from the ranking analysis due to the unavailability of pre-crisis period data, as the indicator was developed at the onset of the COVID-19 pandemic. Each score for every indicator within each resilience domain was aggregated for each country, and the total was divided by the possible maximum score for that country, accounting for the number of indicators with missing data. In cases where two or more countries had the same value for a particular indicator, they were assigned the same rank number. Further details on the methodology can be found in Appendix A.

## 3 Results

### 3.1 Exposure to shocks

#### 3.1.1 COVID-19

**Figure 1:**
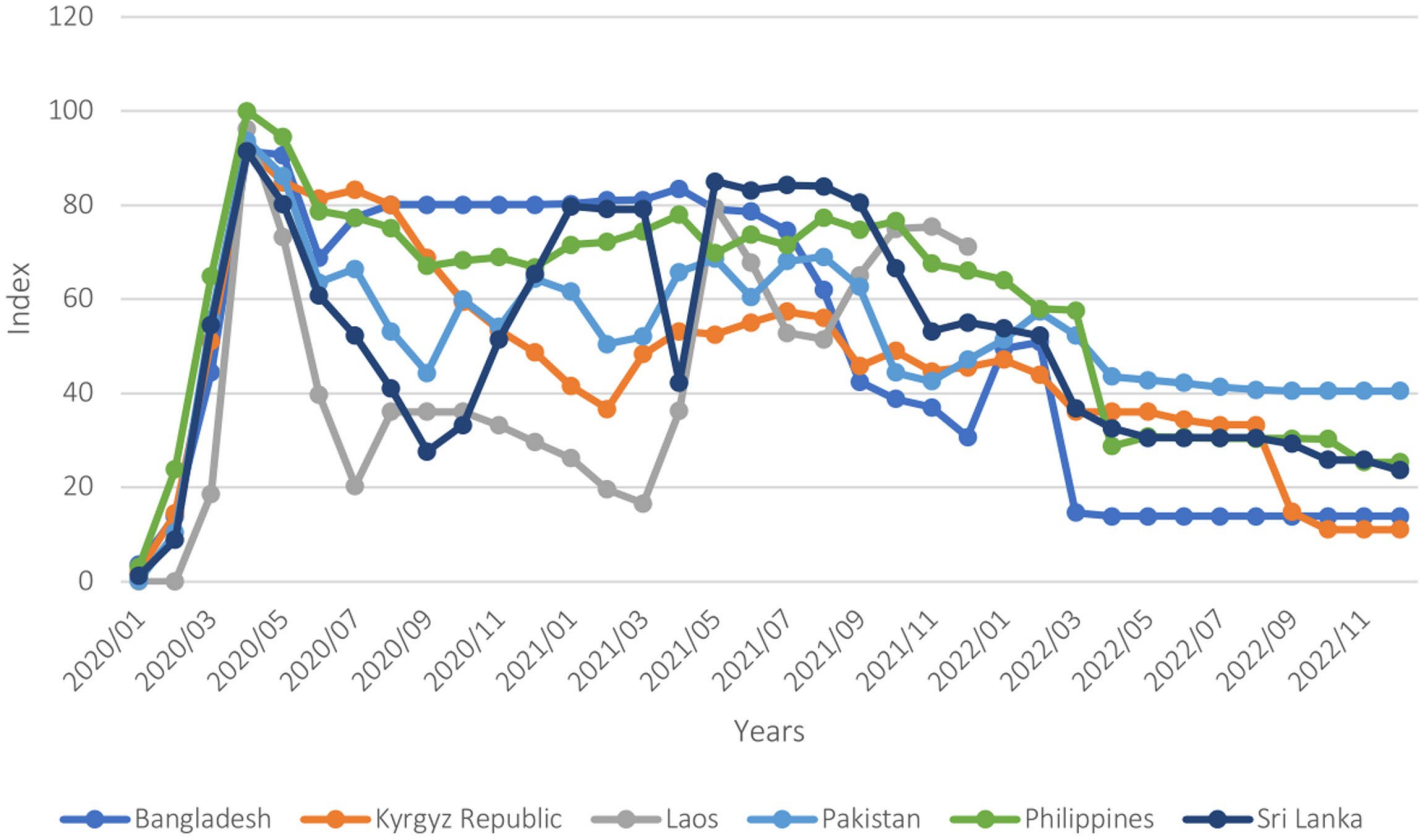
Covid-19 stringency index (January 2020–December 2022). The stringency index is a composite measure based on nine response indicators including school closures, workplace closures, and travel bans, rescaled to a value from 0 to 100 (100 = strictest).

COVID-19 was a major shock felt by all six countries. The COVID-19 Stringency Index (Figure 1) indicates that, while containment measures by governments were promptly and uniformly adopted across all countries to minimize the spread of COVID-19, how stringent and how long these measures were maintained varied. The Philippines and Bangladesh, although they relaxed their stringent measures after a few months from the beginning of the pandemic, maintained relatively high levels of stringency for an extensive period before gradually lifting them. In contrast, Sri Lanka and Lao PDR rapidly began to ease restrictions, adapting their degree of stringency according to transmission levels.

#### 3.1.2 Economic stability

**Figure 2:**
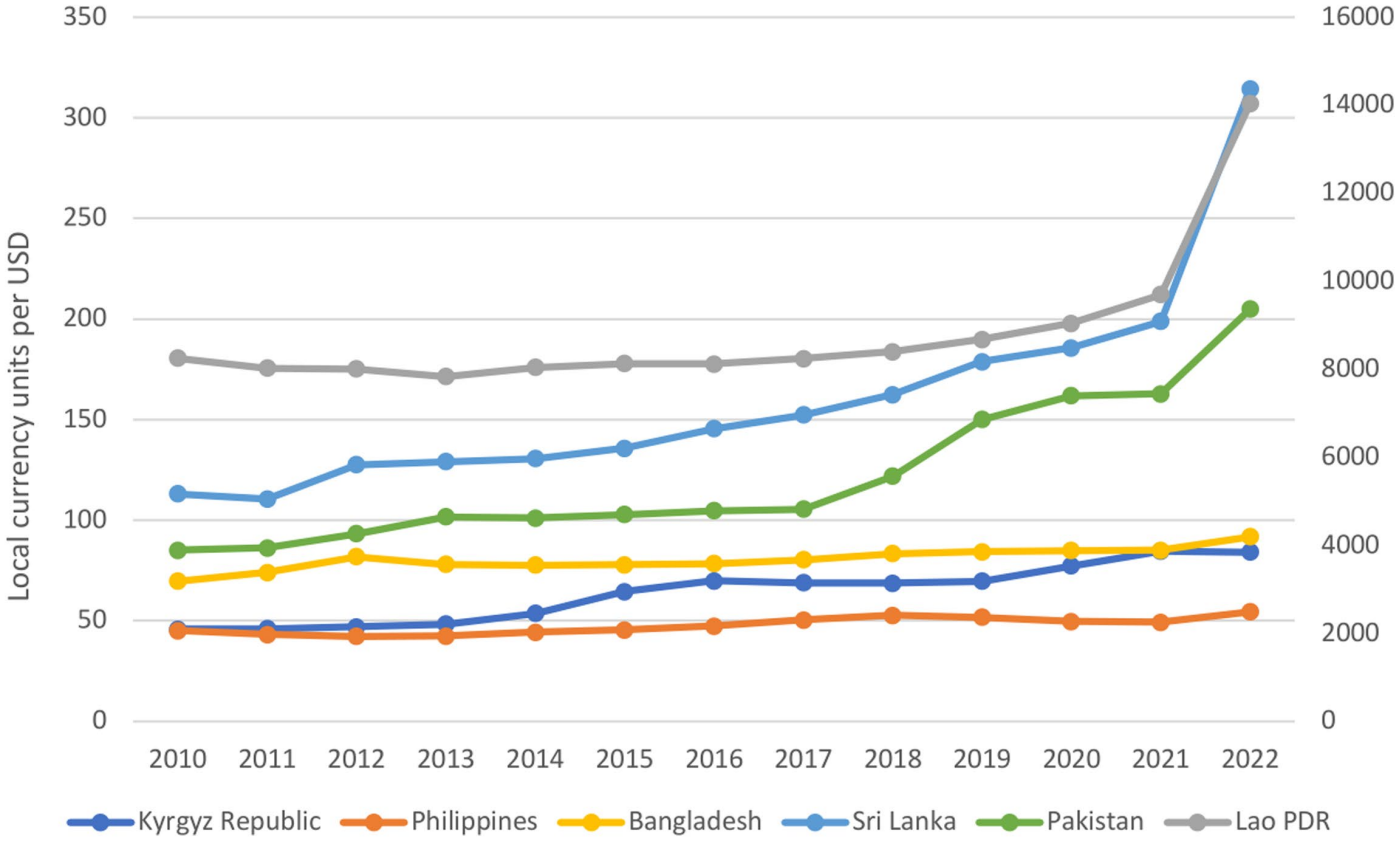
Exchange rate (2010–2022). See the primary vertical axis (0–350 local currency unit per US dollar) for Kyrgyz Republic, Philippines, Bangladesh, Sri Lanka and Pakistan. See the secondary vertical axis (0–16,000 local currency units per US dollar) for Lao PDR.

Significant disparities in exchange rates became evident across the countries (Figure 2). During the crisis period, the depreciation of the Pakistani currency occurring in 2018–19 persisted in 2020, leveled off in 2021, and then increased sharply by 26% in 2022. The Sri Lankan currency exhibited a steady but moderate depreciation during the pre-crisis period and the first 2 years of the current crisis (2020, 2021). However, in 2022, the depreciation of the Sri Lankan currency accentuated, reaching 58%. Similarly, the Lao PDR currency a slightly dropped in value in 2020–21, followed by sharp depreciation, falling by 44% in 2022. In comparison to other countries, the Kyrgyz Republic sustained a moderate depreciation, experiencing a 21% decrease from 2019 to 2022. Bangladesh and the Philippines exhibited more stable trends in their currencies, with the currency of Bangladesh depreciating by 8% and that of the Philippines by 10% only in 2022.

#### 3.1.3 Natural disasters

In 2021, Typhoon Rai affected 11% of the Filipino population. Pakistan faced major floods in 2010 and 2022, impacting 10 and 14% of the Pakistani population, respectively. Sri Lanka confronted floods in 2011 and 2014, along with severe droughts in 2012, 2014, 2016, and 2017. In 2022, almost 5% of the Bangladeshi population was affected by damages from cyclones and floods. Lao PDR experienced adverse effects from floods and storms, particularly in 2013 and 2018. The Kyrgyz Republic was the only country where the ratio of affected people to the total population was nearly zero.

### 3.2 Resilience capacities and agro- and food-diversity

#### 3.2.1 Domestic food production

**Figure 3:**
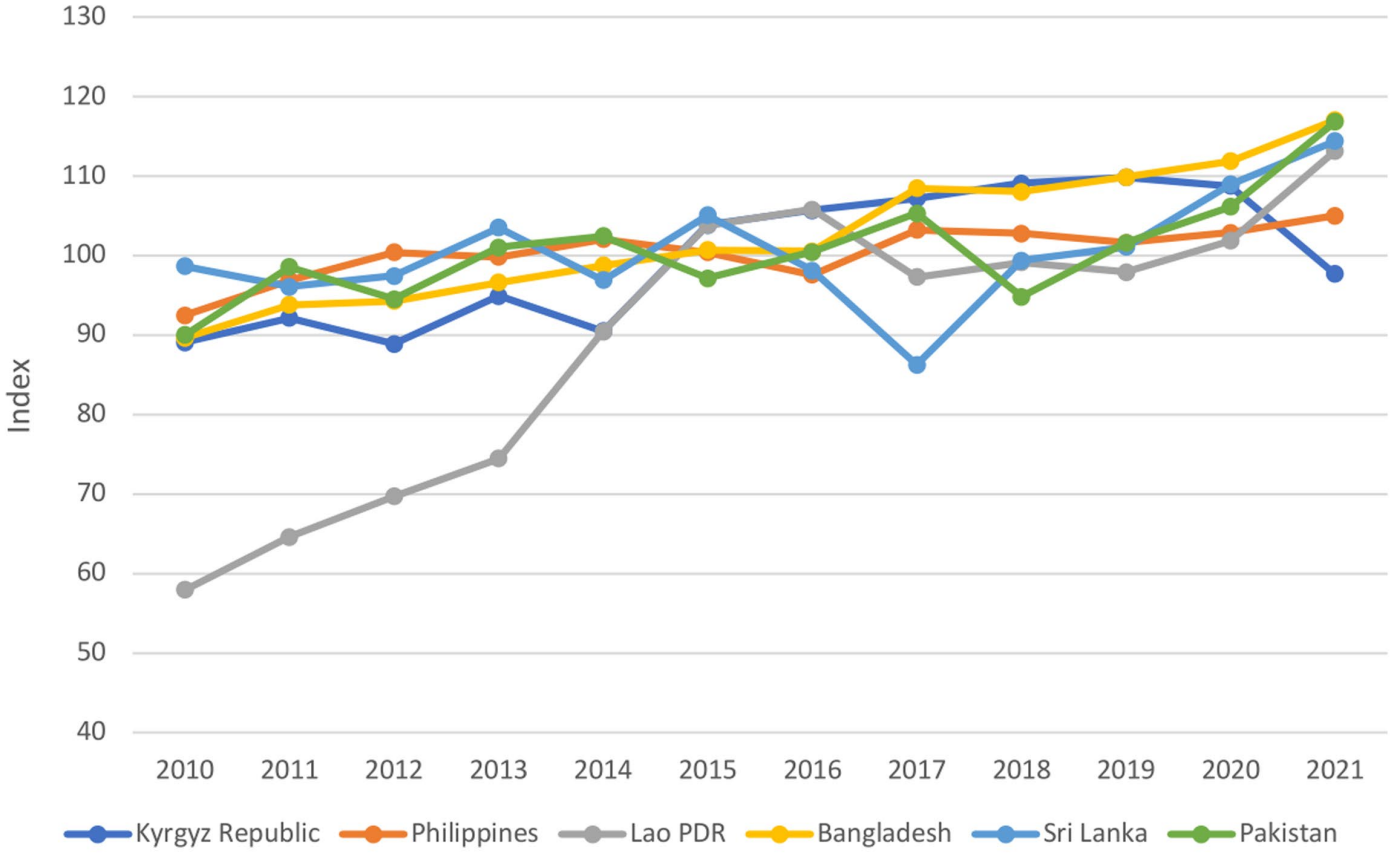
Crop production index (2010–2021). Crop production index shows agricultural production for each year relative to the base period 2014–2016.

The domestic crop production index revealed an increase in crop production from 2019 to the crisis period in all countries except the Kyrgyz Republic, where the index dropped from 110 points in 2019 to 98 points in 2021 (Figure 3). The increase in domestic crop production was mirrored by an increase in fertilizer consumption (Figure A in Appendix B).

The livestock production index showed a similar pattern, however a significant reduction in livestock production was observed in the Philippines (Figure B in Appendix B).

#### 3.2.2 Food imports

**Figure 4:**
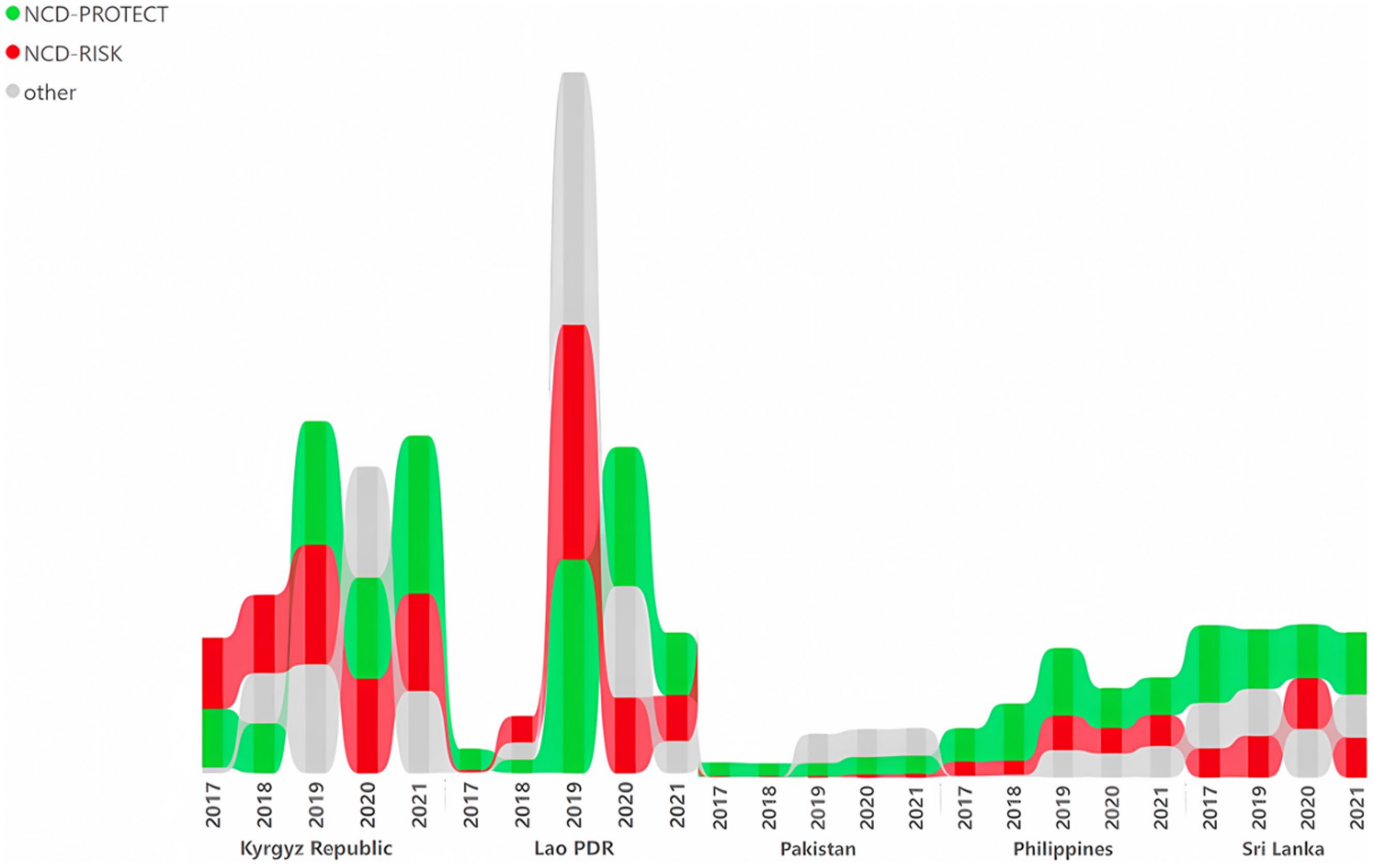
Total *per capita* volume (kilograms or liters) of imports of GDR-classified NCD-risk factor and-protect food groups. NCD-Protect: foods protective against noncommunicable diseases (whole grains; legumes/pulses; vitamin A-rich orange vegetables; dark green leafy vegetables; other vegetables; vitamin A-rich fruits; citrus; other fruits; nuts and seeds). NCD-Risk: foods related to noncommunicable diseases (baked/grain-based sweets; other sweets; processed meat; unprocessed red meat - ruminant; unprocessed red-non ruminant; packaged ultra-processed salty snacks; instant noodles; sugar-sweetened beverages). Other: white roots, tubers, and plantains; eggs; cheese; yogurt; poultry; fish and seafood; Other: 25 Fluid milk; Sweet tea / coffee / cocoa; Fruit juice and fruit-flavored drinks.

Overall, the volume of food imports declined for five of the six countries with available data between 2019 (pre-crisis year) and 2021 (crisis year). At the same time, the volume of GDR-classified NCD-risk foods remained stable over the crisis period for most countries (Figure 4). Kyrgyz Republic, Lao PDR and Sri Lanka had the highest import volumes of NCD-risk food and beverages during the crisis years of the countries studied: in 2021, the Kyrgyz Republic recorded an import of 101 kg of food per 1,000 persons, while Sri Lanka registered 44 kg of food imports per 1,000 persons. In Lao PDR, the import volume of NCD-risk food and beverages was 78 kg per 1,000 persons in 2020, experiencing a decrease to 48 kg per 1,000 persons in 2021. Sri Lanka’s NCD-risk import figures were high, considering that an import ban (Extraordinary Gazette No 2167/10, 2020) was as imposed in the country starting in 2020 in the wake of the COVID-19 pandemic which limited the import of thousands of food and non-food items.

In order to support the findings on food import volumes, we also examined food sales between 2017 and 2023, which mirrored the import findings. The Philippines had by far the highest sales volume of NCD-risk food and beverages across all years, indicating concern for the nutritional quality of diets. In particular, the sales volume of sugar sweetened beverages (SSB), increased in the Philippines, Kyrgyz Republic, Pakistan, and Lao PDR (Figure C in Appendix B).

#### 3.2.3 Infrastructure level and social capital

Overall, social capital, measured by the social capital index, and infrastructure level, measured by mobile cellular subscriptions, remained relatively stable in all countries (Figures D,E in Appendix B).

#### 3.2.4 Economic support

**Figure 5:**
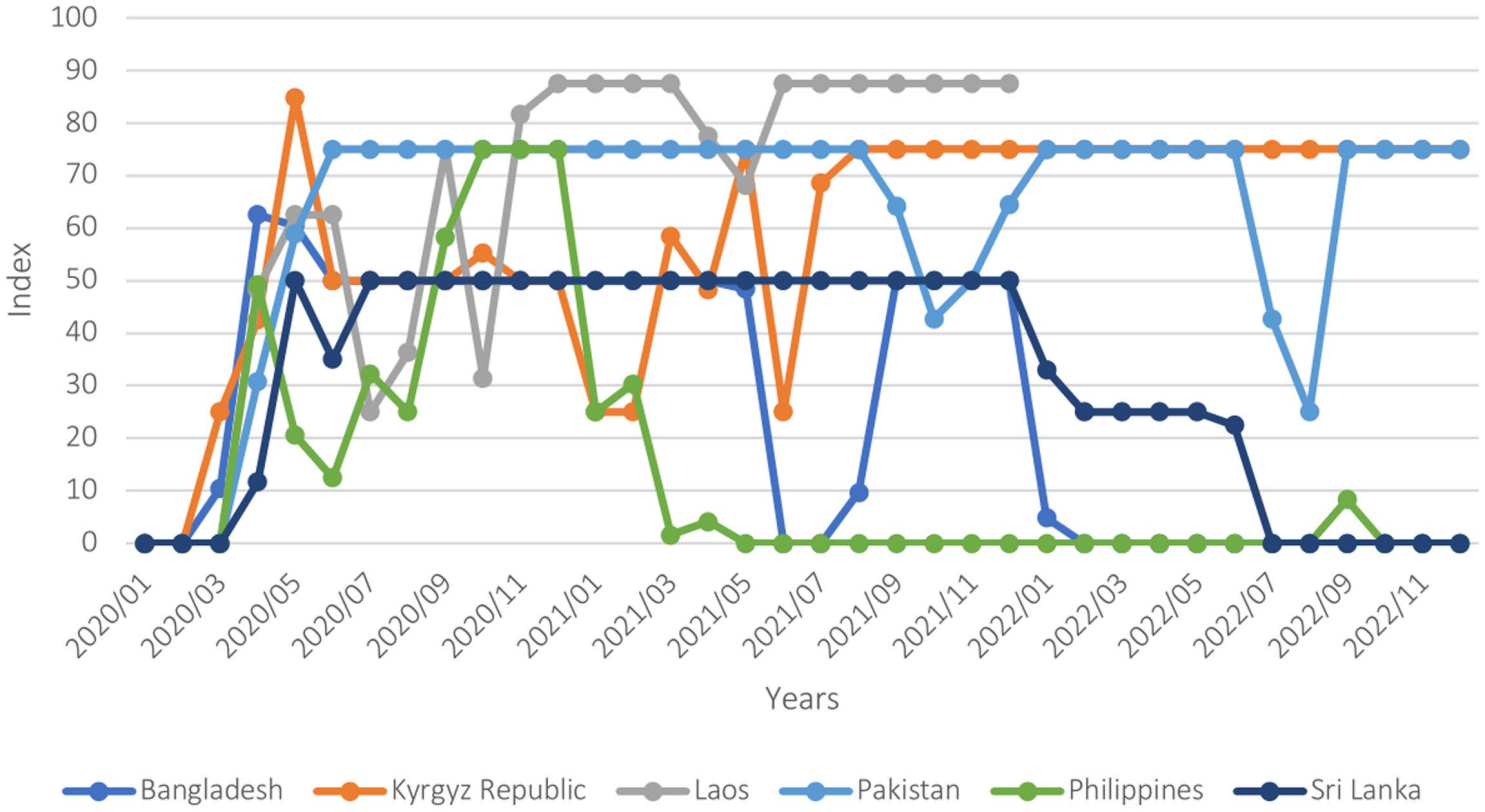
Covid-19 economic support index (January 2020–December 2022). The economic support index is a composite measure based on four indicators: direct transfers to people not working due to the pandemic; debt relief for households; fiscal spending to stimulate the economy; and international support. It is rescaled to a value from 0 to 100 (100 = highest).

The Economic Support Index (Figure 5) shows that various levels of economic support were provided by the governments of the six countries 2 or 3 months after the beginning of the pandemic. The Kyrgyz Republic and Pakistan, despite some fluctuations over time, maintained high levels of economic support throughout the entire year of 2022. Lao PDR exhibited a similar trend, but with economic support decreasing to zero in October 2022 and then rebounding the following month, whereas the Philippines provided moderate support during the early months of the pandemic, increased its support to a high level for a few months in late 2020 and quickly reduced it to minimal levels in early 2021.

### 3.3 Resilience responses and strategies

#### 3.3.1 Coping strategies

**Figure 6:**
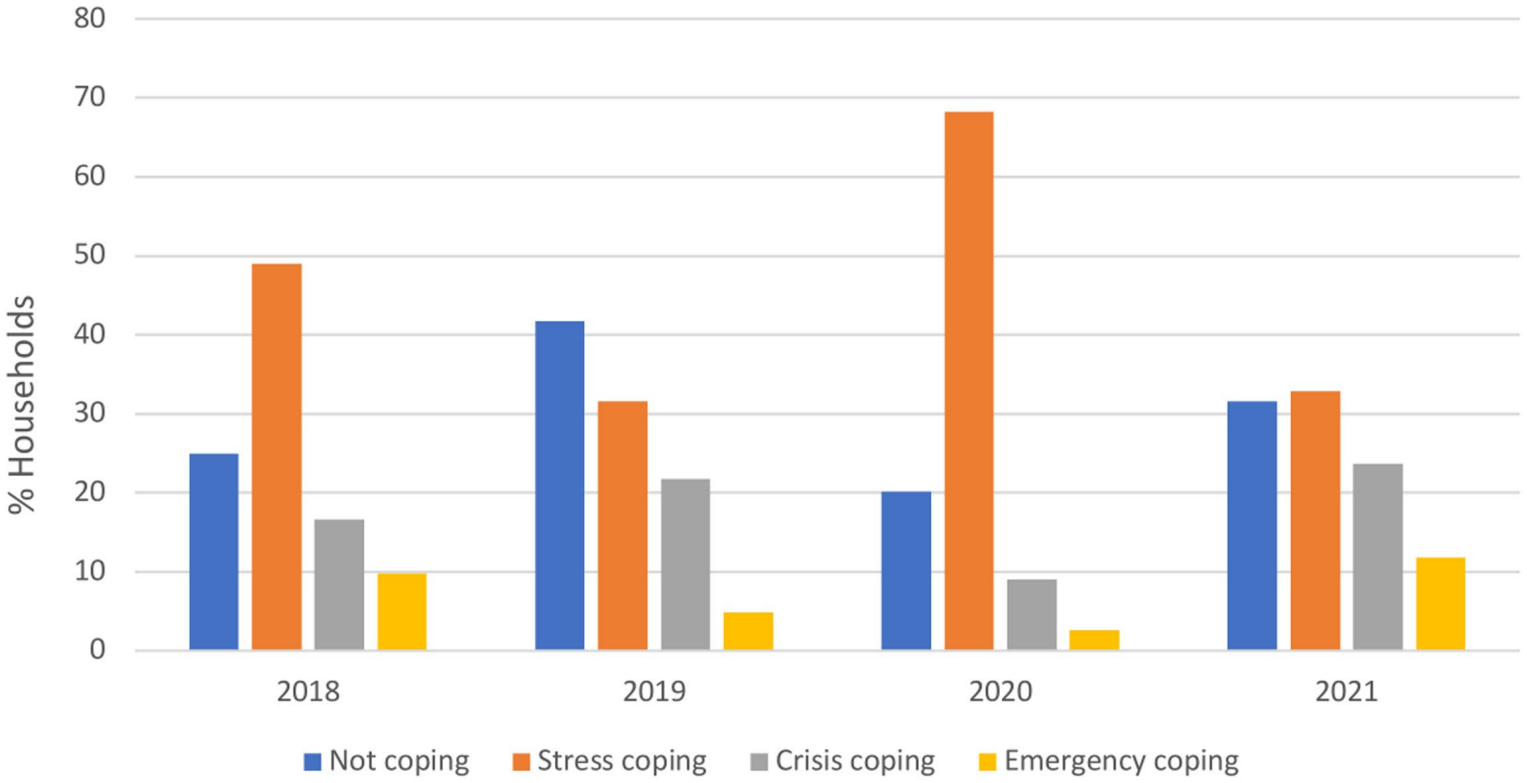
Proportion of households relying on livelihood coping strategies for food security in Kyrgyz Republic from 2018 to 2021. Stress coping strategies: indicates a reduced ability to deal with future shocks due to a current reduction in resources or increase in debts. Crisis coping strategies: directly reduces future productivity, including human capital formation. Emergencies coping strategies: affects future productivity but are extremely difficult to reverse or more dramatic in nature.

**Figure 7:**
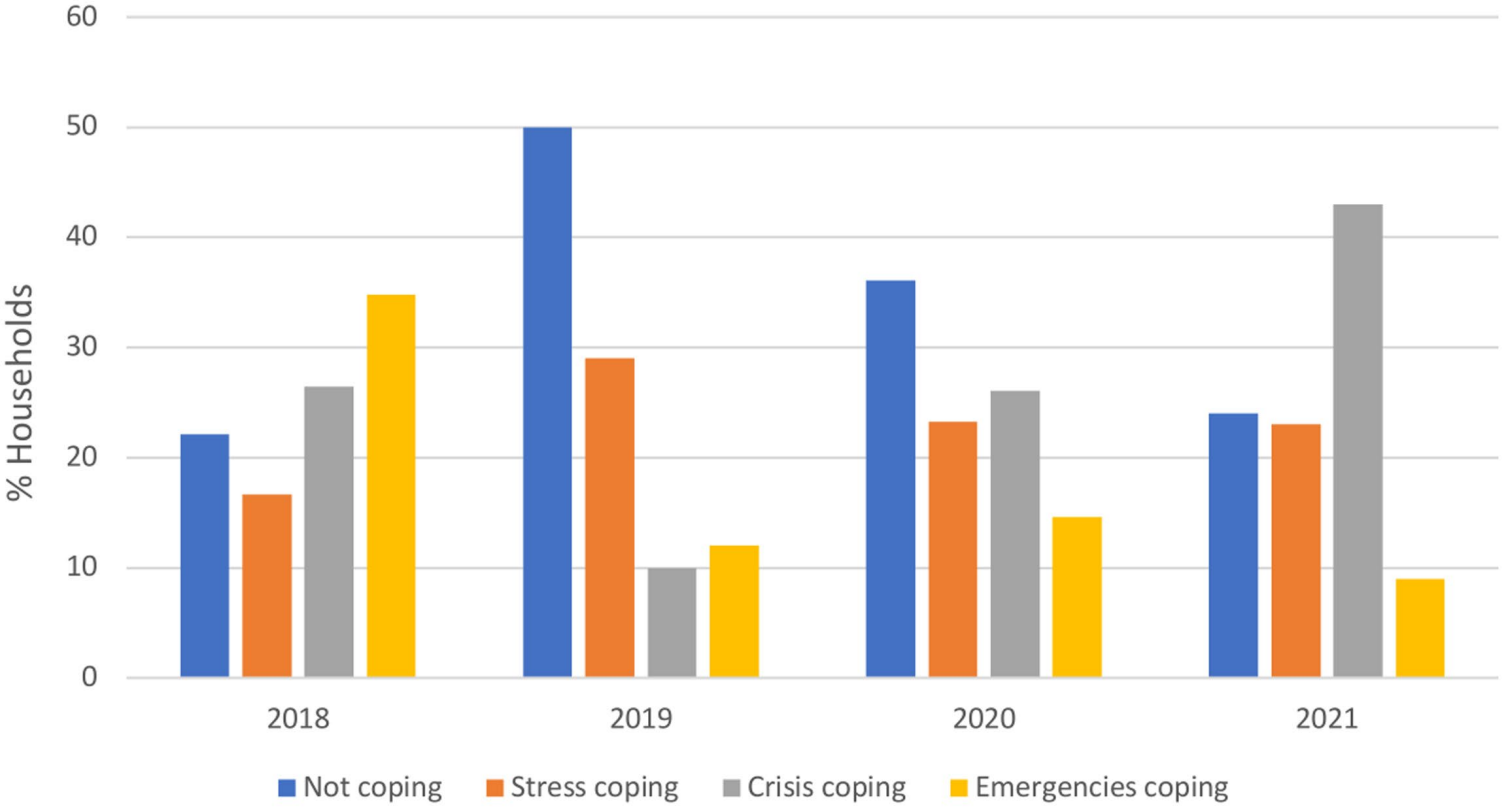
Proportion of households relying on livelihood coping strategies for food security in Pakistan from 2018 to 2021. Stress coping strategies: indicates a reduced ability to deal with future shocks due to a current reduction in resources or increase in debts. Crisis coping strategies: directly reduces future productivity, including human capital formation. Emergencies coping strategies: affects future productivity but are extremely difficult to reverse or more dramatic in nature.

Kyrgyz Republic and Pakistan were the only countries with available pre- and during-crisis data for trend analysis of coping strategy indicators. In the Kyrgyz Republic, the national average rCSI increased from a mean score of 3.8 in 2019 to 8.2 in 2021, indicating that households relied more frequently on extreme food-based coping strategies (e.g., limiting portion size at mealtime; Supplementary Table). Regarding LCS-FS, the use of stress coping mechanisms increased in 2020 (reported by 68% of households) but returned to pre-crisis levels in 2021 (reported by 33% households; Figure 6). The use of crisis and emergency coping mechanisms escalated drastically in 2021, although they had declined in 2020. These results reflect an erosion of households’ capacities to withstand shocks and future productivity. In Pakistan, the rCSI showed a decrease in “medium coping” and “high coping” categories and a sharp increase in the “low coping” category in 2020 compared to 2019 (Supplementary Table). Similarly, when looking at LCS-FS, the use of drastic coping mechanisms declined between 2019 and 2020 [from 26 to 10% (crisis) and 35 to 12% (emergency)] and stress coping strategies or not at all increased (Figure 7). However, in 2021 and 2022, there was a slight decline in the use of stress and emergency mechanisms, a notable decrease in those not using any coping strategies, and a significant increase in the use of crisis strategies.

### 3.4 Long-term resilience outcomes

#### 3.4.1 Food price volatility

**Figure 8:**
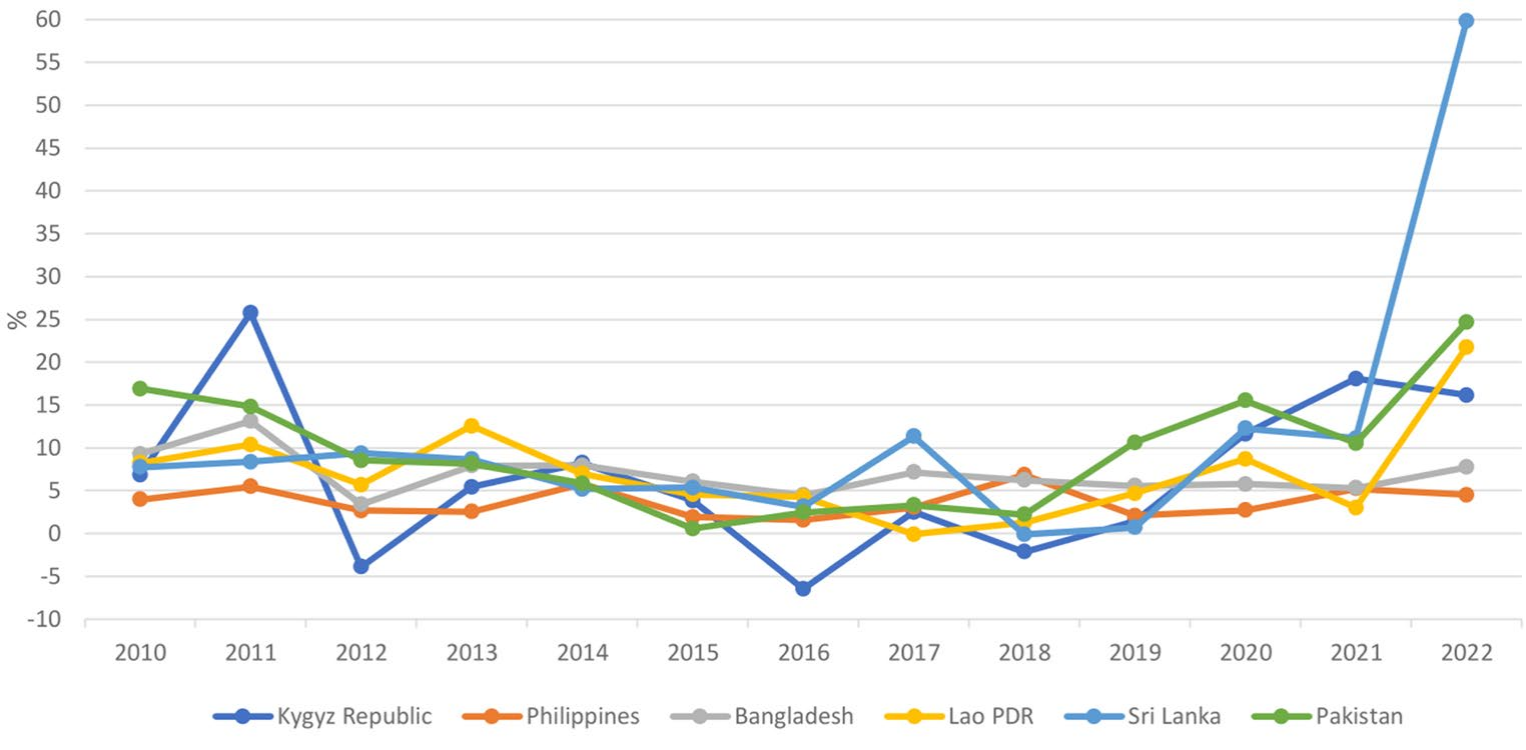
Annual food price inflation (2010–2022).

Food price inflation in the six countries showed an increase from 2019 to the crisis period in all countries, especially in Sri Lanka, where inflation rose to 12.3% in 2020, remained at 11.1% in 2021, and spiked to 59.8% in 2022 (Figure 8).

Analysis of food price anomalies for rice from 2015 to 2022 highlighted differences in price stability, with Bangladesh, Pakistan, and the Philippines maintaining relatively steady rice prices, while Lao PDR and Sri Lanka experienced moderate to high price growth (Figures F–H in Appendix B).

#### 3.4.2 Food security

**Figure 9:**
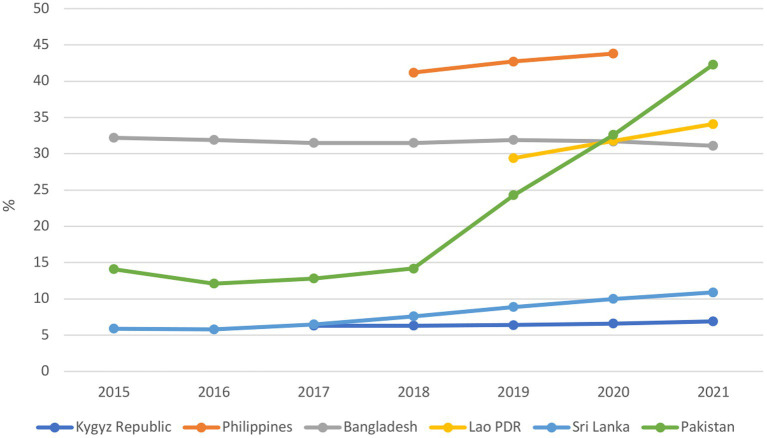
Percent of the population experiencing moderate or severe food insecurity.

The prevalence of moderate or severe food insecurity increased slightly from pre-crisis to the crisis period in all countries except Bangladesh, where the prevalence remained stable. Pakistan had the most dramatic increase in food insecurity, from 14.2% in 2018 to 42.3% in 2021 in Pakistan (Figure 9). Similarly, there was an increase in the percentage of the population unable to afford a healthy diet from 2019 to the crisis period, with the exception of Bangladesh, where a declining trend was maintained (Figure I in Appendix B).

In order to support the findings on food security, we also examined the cost of diet. For all countries with available data, again with the exception of Bangladesh, there were greater increases in the cost of a nutritious diet compared to an energy-only diet. For Lao PDR, Sri Lanka and the Philippines, significant increases in the median cost of a nutritious diet were observed. For example, the median cost of a nutritious diet in Sri Lanka is nearly 300 percent higher compared to an energy-only diet (Figures J,K in Appendix B).

**Figure 10:**
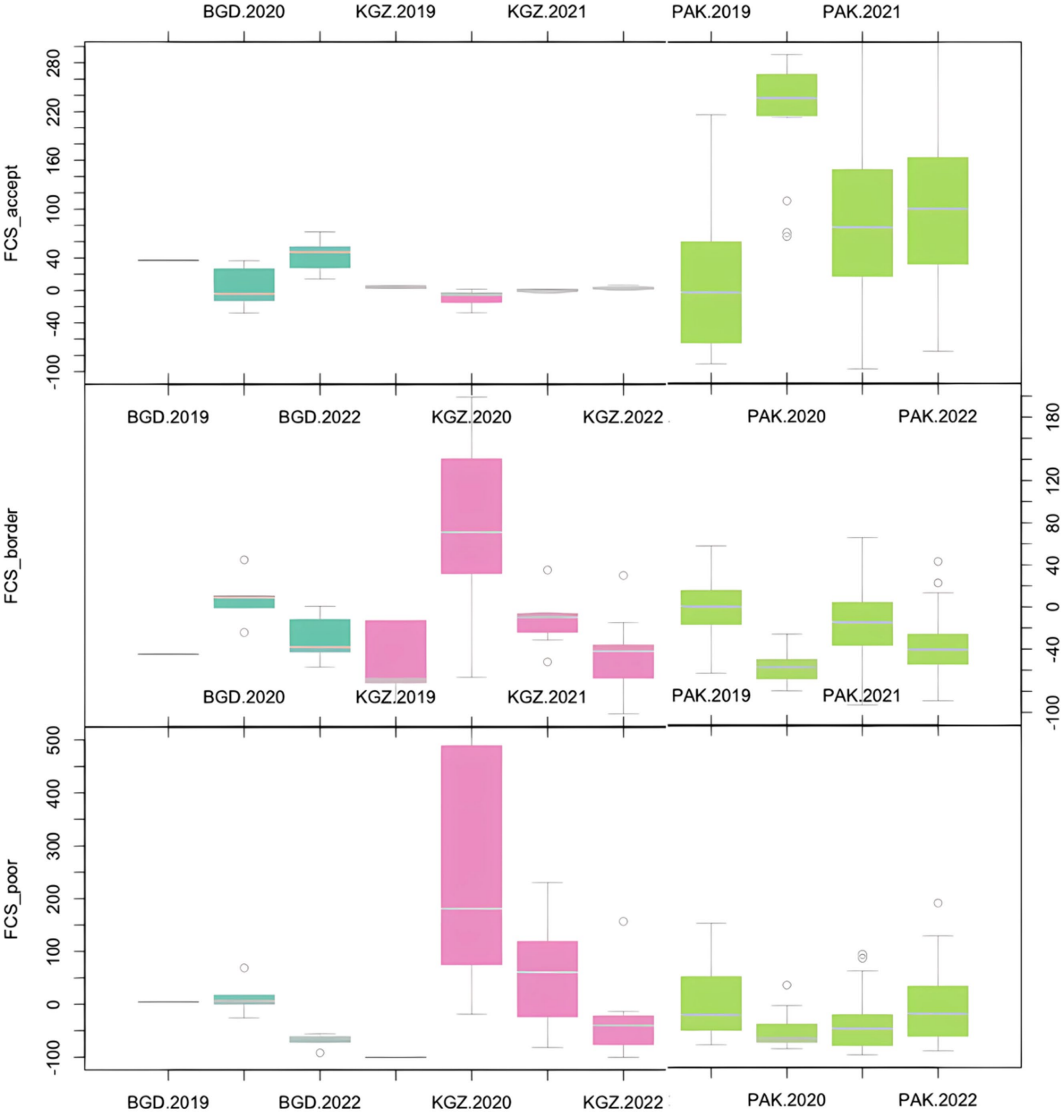
Percent change in household food consumption scores for three countries (baseline to 2022). BGD, Bangladesh; KGZ, Kyrgyz Republic; PAK, Pakistan. FCS_accept: households with acceptable Food Consumption Score (>42); FCS_border: households with borderline Food Consumption Score (28.001–42); FCS_poor: households with poor Food Consumption Score (≤28).

The analysis of percent change in FCS from the baseline point (2019) for Bangladesh, the Kyrgyz Republic, and Pakistan, showed that, in each of these three countries, the median acceptable FCS (FCS > 42) increased from baseline over the course of the crisis (Figure 10). For Pakistan, overall food consumption scores significantly improved in crisis year 2020 before worsening slightly in 2021 and 2022, but remaining above pre-crisis levels, with more households moving from acceptable to borderline (FCS = 28.001–42) scores in 2021. Median acceptable FCS decreased in Kyrgyz Republic and Bangladesh in crisis year 2020 before rising again. However, borderline FCS increased from the baseline in Bangladesh and the Kyrgyz Republic over the course of the crisis, spiking in 2020 then lowering, but remaining above the baseline in 2021 and 2022. Poor FCS (FCS≤28) went down in Bangladesh and Pakistan over the course of the crisis, while rising in the Kyrgyz Republic.

### 3.5 Model predictive power

Lao PDR had both the lowest total resilience capacity and lowest longer term resilience outcomes scores. This trend was also evident for two bottom-ranked countries, Pakistan and Bangladesh. Sri Lanka and Philippines had the highest combined scores for total resilience capacities and agrofood diversity pre-crisis, and the second and third highest scores for longer-term resilience outcomes, respectively. Kyrgyz Republic somewhat buckled the trend with only the third highest resilience capacity score, but by far the highest longer-term resilience outcome score (Table 3).

**Table 3:** Total scores for resilience capacities and agro- and food diversity, and long-term resilience outcomes for each of the six countries.

	Resilience capacities and agro- and food diversity*	Long-term resilience outcomes**
Sri Lanka	0.64	0.58
Philippines	0.64	0.53
Kyrgyz Republic	0.58	0.67
Bangladesh	0.57	0.50
Pakistan	0.56	0.44
Lao PDR	0.37	0.43

*Calculated for the following indicators: Crop production index, Fertilizer consumption, Livestock production index, Food import, NCD-protect, Mobile Cellular subscription, and Social capital index. **Calculated for the following indicators: Food Price Inflation, Food Price Anomalies (IFPA), by type of product (Rice), Food Price Anomalies (IFPA), by type of product (Wheat), Food supply variability, % population experiencing moderate or severe food insecurity, % population who cannot afford a healthy diet.

## 4 Discussion

The unfolding global polycrisis has revealed a web of vulnerabilities within food systems and the findings of this study provide valuable insights into the diverse challenges and resilience capacities within each of the six countries - Bangladesh, Kyrgyz Republic, Lao PDR, Pakistan, Philippines, and Sri Lanka. The analysis of resilience capacities and agro-and food-diversity revealed varying growth rates. For resilience outcomes, the study revealed fluctuations in national food price inflation patterns, rice price anomalies, and the percentage of the population unable to afford a healthy diet across the countries. The findings underscored the challenges and adaptive capabilities within each country, shaped by economic, political, agricultural, and food affordability dynamics contributing to the long-term resilience of their food systems.

Despite differences in the set of challenges faced by the selected countries, there were some regional commonalities which are important for understanding and addressing food system resilience. The first characteristic is the Asia-Pacific region’s extreme susceptibility to climate events brought about by climate change. The World Risk Index, which ranks countries based on their vulnerability to extreme weather events, lists seven Asia-Pacific countries among the top 10 countries with the highest risk (22). This expected increased frequency of climate-related events, including floods, landslides, droughts, and storms poses a severe test to the resilience of each country’s food system, leading to disruptions in food supply chains, transportation, food price volatility and compromised food security as well as changes in yield, biomass, food composition, and nutritional quality, directly influencing human nutrition and health (23).

The second characteristic is the region’s reliance on imports for much of its food supply. Most countries in the region are net food importers, often from the Ukraine, Russia, or both, leaving them “highly exposed” to rising global food prices (24). The third is the rapid and unprecedented urbanization taking place, referred to as “the defining megatrend” in the region (25), with nearly 55% of the region’s population expected to live in urban areas by 2030 (26).

Our comparison of total scores for resilience capacities pre-crisis and total scores for long-term resilience outcomes measured in 2022 showed a tendency for countries with a higher total resilience capacity score at the outset of the polycrisis to also be the countries with a higher score for long-term resilience outcomes. In other words, countries with higher total resilience capacities exhibited more desirable long-term resilience outcomes compared with countries with lower resilience capacities. Regarding resilience capacities and agro-food-diversity, food production increased from the pre-crisis starting point at a more accelerated pace than previous years in the majority of countries, demonstrated considerable strength in coping with successive shocks. This finding aligns with a study conducted by Dixon et al. (27) on the response and resilience of Asian agri-food systems to COVID-19. Their research highlighted the overall resilience of smallholder Asian farming food systems, which constitute a significant portion of the agricultural workforce in Asia (28), during the pandemic. Rural areas, especially in the countries targeted in our study, exhibited greater resilience due to lower population densities, resulting in slower coronavirus transmission compared to urban areas. These countries showed resilience, with smallholder farms being relatively diversified, maintaining significant self-sufficiency (though declining), and having access to local markets for various farm and household needs, except during periods of obligatory closure, lockdown, or supply chain disruption.

Regarding long-term resilience outcomes, no country was immune from substantial food price inflation during the crisis period, reaching historic highs. A link between disruption in food supply caused by COVID-19 and food price inflation and has been documented by others (29). Unlike previous major food price spikes in 2007–2008 and 2010–2012, this time, inflation was correlated with the depreciation of countries’ currencies against the US dollar (30). The volatility of food prices was also evident in the observed price anomalies for rice, the most important staple food in Asia, across all countries. Food security indicators worsened proportionally from pre-crisis to the crisis period, i.e., countries with the highest pre-crisis food insecurity still experienced the highest food insecurity in the crisis period. Several reasons for the increase in food insecurity in the region have been documented, including COVID-19 leading to disruptions in national and international food supply chains, affecting the availability and accessibility of food items (31, 32). Furthermore, lockdowns resulted in immediate job losses, causing a reduction in household income (33), and the ramifications of the war in Ukraine, coupled with the rapid acceleration of food inflation, further exacerbated household food insecurity, reducing purchasing power (34). As a result, households scaled up their own-food production and foraging activities, reduced their food consumption, or shifted to cheaper foods, potentially resulting in inadequate nutritional intake. However, it is important to note that our resilience outcome data only extended to 2021, reflecting only the impact of the shock caused by COVID-19 and not the war in Ukraine which began in 2022.

Looking beyond regional characteristics and the overall association between resilience capacities and long-term resilience outcomes, the selected countries handled the impacts of the shocks (Covid-19 and the war in Ukraine) differently. This was influenced by their unique food system contexts before the onset of the shocks, as well as their internal economic and political situation.

Lao PDR, consistently exhibiting the lowest scores in resilience capacities and agro- and food diversity, as also recorded the lowest score in long-term resilience outcomes. Despite fluctuations in recent years, it consistently had the lowest crop production index in the years preceding the crisis among the six countries. This negative trend can be attributed to the country’s predominantly traditional and less productive agricultural practices (35), as well as its high vulnerability to climate-related hazards, particularly floods and droughts (36). In an already weak food system heavily reliant on agriculture, especially for rural communities, the recent shocks have further deteriorated resilience outcomes and increased food insecurity, which was already one of the highest among the countries.

Over the years, Pakistan has faced drought emergencies, resulting in severe water shortages that reduced irrigated land and crop production (37). This contributed to a low score in agro- and food diversity capacities. The country’s food system also exhibits high volatility, especially in the unstable price of wheat, exacerbated by the drop in grain exports due to the war in Ukraine (38). Limited food storage capacity at household and governmental levels further weakens resilience, impacting the purchasing power and food access for the population (39). Even before the crisis, a significant proportion of the population struggled to afford a healthy diet, worsening in 2020 and making Pakistan face the worst food security outcomes among the targeted countries.

Bangladesh showed high scores for crop production and fertilizer consumption, yet low scores for livestock production, infrastructure, and social capital. In comparison to other countries where food supply variability received the lowest score, Bangladesh maintained constant values. This suggests the food system’s ability to sustain constant variability in food supply in the face of shocks. Notably, Bangladesh is the only country where a decrease in the percentage of the population unable to afford a healthy diet was observed. An IFPRI study (40) reported a reduction in food insecurity to pre-pandemic levels, possibly attributed to the relaxation of COVID-19 restrictions and subsequently resumption of job activities.

Although the Kyrgyz Republic did not exhibit the highest resilience capacity score, mainly due to its much lower fertilizer consumption compared to other countries, it showed the highest long-term resilience outcome score. This was mainly thanks to the proportion of the population experiencing moderate or severe food insecurity, which has remained constant and at a low level, even during crisis periods. Nevertheless, these numbers face increasing threats from growing vulnerabilities to substantial shocks and stress due to anticipated climate change (41). The reliance on domestic water supply from glaciers and snowmelt for agriculture, coupled with significant water losses from aging infrastructure, poses a significant concern for the country’s food system (42).

The Philippines, standing out with the highest scores in resilience capacities alongside Sri Lanka, demonstrates high capacities linked to infrastructure and social capital, despite challenges such as low livestock production both before and during the crisis period. This challenge is attributed to the impact of epidemics such as avian flu and swine flu on food systems, leading to shortages of key food items (43). Interestingly, despite large variations in food inflation experienced by all countries, the Philippines was the least affected. However, it recorded the highest number of food-insecure people, particularly in Quezon City, emphasizing the significant challenges posed by rapid urbanization on the city’s food system during a crisis, as revealed by a study conducted by Auma et al. (44).

Sri Lanka demonstrated generally high capacities in agro- and food supply diversity, as well as in infrastructure and social capital. However, the country received the lowest score for food inflation due to skyrocketing food prices, significantly impacting the population’s purchasing power and access to food (45). According to a FAO report, factors contributing to the increased prices include low market availability, high production and transport costs, and disruptions in marketing activities due to severe fuel shortages (46). An additional factor influencing the heightened prices was the government-imposed ban on chemical fertilizer imports in April 2021, leading to a substantial reduction in rice harvests and subsequently driving up food prices (47). Nevertheless, Sri Lanka still exhibited the lowest number of people who could not afford a healthy diet compared to other countries, indicating a certain resilience in its food system.

This study has several important strengths. Firstly, it employed a conceptual model that provided a comprehensive assessment of food system resilience across six diverse countries. The model considered various dimensions and interrelated factors influencing food security and nutrition, providing a holistic view of the complex dynamics within each country’s food system at the national level. The inclusion of interviews with representatives from WFP country offices was another strength, enabling us to contextualize the data extracted from global data sources and gain deeper insights into the food systems of each targeted country. Additionally, we could access unpublished information on food security and food resilience, offering an idea of what to expect in the near future in terms of food system resilience indicators. Furthermore, transformation of food trade data was conducted to examine the data through nutritional lenses. This approach allows us not only to investigate trends in food imports and assess how they have been affected by the crisis but also to distinguish the impact on healthy food and foods whose consumption should be limited.

Certain limitations should also be considered when interpreting our findings. Utilizing national level data does not capture the nuanced disparities and vulnerabilities faced by specific population segments, particularly lower-income households, minority groups, displaced persons, women and children, who are likely to be disproportionally affected by the polycrisis. This limitation hinders a comprehensive understanding of how these inequalities affect resilience and adaptive capacities within food systems and points to a need for more localized and granular data in order to effectively target those most in need. Additionally, annual averages might mask stronger fluctuations in data that some indicators might have experienced during the year, and which could be captured with more frequently collected data. There were also substantial data gaps over the study period, especially for 2022 and 2023, meaning that the trends of some indicators may not have captured the effects of the war in Ukraine that started in 2022. This hampered the ability to provide an accurate assessment of the ongoing crisis’s evolving nature and full effects on food systems. Furthermore, for indicators related to food security, for which data were supplied by the WFP, data collection methodologies and sampling methods vary among countries. In some instances, data were only collected from selected provinces in a country. Therefore, the presented results may not be wholly representative of the entire country. Finally, the conceptual model does not fully encapsulate the temporal dynamics of the crisis, as evolving impacts might not be fully captured in the reviewed data. Understanding the long-term effects of the crisis on food systems is challenging within the study’s current scope.

### 4.1 Recommendations for future research

#### 4.2 Resilience assessment in food systems

Research should delve into identifying which indicators prove most descriptive and useful across different levels and components of the food system and identify the minimum set of indicators that can be used to measure the entire food systems continuum (13, 48). These should be relevant for and applicable to diverse food systems at both national and subnational levels. Understanding the determinants of resilience is equally crucial, prompting an investigation into the factors that contribute to variations in resilience. This multi-dimensional analysis, encompassing socio-economic, environmental, and institutional dimensions, can provide a nuanced comprehension of the diverse pathways to food system resilience. Research should also consider the potential prolonged impact on food systems and their adaptive capacities beyond the immediate crisis phase.

#### 4.3 Integration of climate change adaptation

To fortify food systems against the backdrop of climate change, it is imperative to gage the extent of integration of adaptation measures (49, 50). Research should scrutinize the degree to which countries have embedded climate change adaptation strategies into their food systems and assess the impact of these measures during extreme weather events. This line of inquiry will shed light on the effectiveness of specific adaptation measures, offering valuable insights for refining strategies that enhance resilience.

#### 4.4 Early intervention strategies

The anticipation and management of impending food system shocks represent a critical dimension of resilience (51). Research endeavors should focus on identifying emerging trends or indicators that act as precursors to potential shocks. This exploration could pave the way for the development of predictive models, offering a proactive approach to early intervention strategies including anticipatory actions. Investigating the effectiveness of these models in real-world scenarios can significantly contribute to refining and tailoring strategies for timely responses to imminent challenges.

## 5 Conclusion

This study on the resilience of food systems in six Asia-Pacific countries amid global crises highlights the complex challenges posed by COVID-19, geopolitical conflicts, and climate change. It reveals disparities in resilience across countries, shaped by economic, political, and agricultural factors. The paper underscores the need for adaptive governance, targeted policy interventions, and transformative changes in food system management. The findings advocate for future research on resilience indicators, climate change adaptation, and early intervention strategies to strengthen food systems against global challenges.

## Data availability statement

The original contributions presented in the study are included in the article/Supplementary material, further inquiries can be directed to the corresponding author.

## Author contributions

CF: Writing – original draft, Writing – review & editing. CC: Formal analysis, Writing – review & editing. EW: Writing – original draft. KS: Writing – review & editing. MM: Writing – review & editing. DC: Formal analysis, Writing – review & editing. AS: Supervision, Writing – review & editing. JK: Supervision, Writing – review & editing. SM: Supervision, Writing – review & editing. SG: Supervision, Writing – original draft.
