# Supplementary material for: Exploring food system resilience to the global polycrisis in six Asian countries
Source: Front Nutr. 2024 Apr 16;11:1347186. doi: 10.3389/fnut.2024.1347186 (PMC11059780; doi:10.3389/fnut.2024.1347186)
Supplement: Supplementary file 2 [file Table_2.PDF]

# *Supplementary Material*

## Appendix A: Methods

### A.1 – Categorizing food trade for nutrition analysis

The table below shows the classification of DQQ1 food groups into three GDR[2] food categories: "NCD[3] protect" foods, serving to protect against NCDs, "NCD risk" foods, known to pose a risk for NCDs, and "other foods" group, including food items classified as neither NCD-risk nor NCD-protect.

| **NCD Protect Foods** and NCD Risk Factor Foods | |
|---|---|
| 1 Foods made from grains | 16 Processed meat |
| 2 **Whole grains** | 17 Unprocessed red meat (ruminant) |
| 3 White roots, tubers, and plantains | 18 Unprocessed red meat (non-ruminant) |
| 4 **Legumes/Pulses** | 19 Poultry |
| 5 **Vitamin A-rich orange vegetables*** | 20 Fish and seafood |
| 6 **Dark green leafy vegetables*** | 21 **Nuts and seeds** |
| 7 **Other vegetables** | 22 Packaged ultra-processed salty snacks |
| 8 **Vitamin A-rich fruits*** | 23 Instant noodles |
| 9 **Citrus** | 24 Deep fried food ◁ |
| 10 **Other fruits** | 25 Fluid milk |
| 11 Baked / grain-based sweets | 26 Sweet tea / coffee / cocoa |
| 12 Other sweets | 27 Fruit juice and fruit-flavored drinks |
| 13 Eggs | 28 Sugar-sweetened beverages (soft drinks) |
| 14 Cheese | 29. Fast foods ◁ |
| 15 Yogurt | |
| Certain food items were excluded from the analysis, including bottled water, bouillon and stocks, herbs and spices, sauces, pickled products, coffee whiteners, alcohol, water, and certain oils or other fats which were not intended for consumption. | |
| *Due to UN Comtrade's existing food categories in their database, it is not possible to single out commodities that meet these criteria.<br><br>◁ This category is unavailable in UN Comtrade databases. | |

[1] Global Diet Quality Project's Diet Quality Questionnaire
[2] Global Dietary Recommendations
[3] Noncommunicable diseases

**A.2 – Evolution of indicators of 'resilience capacities and agro- and food-diversity' domain from pre-crisis to crisis period in 6 countries.**

| Resilience sub-domain | Indicator | Unit | Data period covered | Desirable direction | Actual direction during crisis* | | | | | |
|---|---|---|---|---|---|---|---|---|---|---|
| | | | | | Bangladesh | Kyrgyz Republic | Lao | Pakistan | Philippines | Sri Lanka |
| Food produced domestically | Crop production index (2014-2016=100) | Index | 2010-2021 | ↑ (blue) | ↑ (green) | ↘ (orange) | ↑ (green) | ↑ (green) | ↗ (light green) | ↑ (green) |
| | Livestock production index (2014-2016=100) | Index | 2010-2021 | ↑ (blue) | ↑ (green) | → (blue) | ↗ (light green) | ↑ (green) | ↓ (red) | ↑ (green) |
| | Fertilizer consumption | Kg/ha of arable land | 2010-2020 | → (blue) or ↑ (blue) | → (blue) | → (blue) | | → (blue) | ↑ (green) | ↑ (green) |
| Imported food | Food import – NCD-protect | Kg/capita | 2010-2021 | ↑ (blue) | | ↑ (green) | ↓ (red) | ↗ (light green) | ↓ (red) | → (blue) |
| | Food import – NCD-risk | Kg/capita | 2010-2021 | ↓ (blue) | | → (blue) | ↓ (green) | → (blue) | → (blue) | → (blue) |
| Infrastructure | Mobile cellular subscription | Number/100 people | 2010-2022 | ↑ (blue) | → (blue) | → (blue) | → (blue) | ↗ (light green) | → (blue) | ↗ (light green) |
| Social capital | Social capital index | Index | 2010-2023 | ↑ (blue) | → (blue) | ↓ (red) | ↗ (light green) | ↘ (orange) | → (blue) | → (blue) |

* Average crisis period compared to pre-crisis average (2018-2019 depending on data availability)
Desirable direction: denotes a higher value is more desirable,  denotes a lower value is more desirable.
Actual direction : a blue arrow denotes no substantial changes and stable value, a green arrow (up/down) denotes a direction similar to the desirable one, a light green arrow (diagonal up/down) denotes a direction similar to the desirable one but less pronounced, a red arrow (up/down) denotes an opposite direction to the desirable one, an orange arrow (diagonal up/down) denotes an opposite direction to the desirable one but less pronounce

**A.2 – Evolution of indicators of ‘resilience responses/strategies’ and ‘longer-term resilience outcomes’ domains from pre-crisis to crisis period in 6 countries.**

| Resilience sub-domain | Indicator | Unit | Data period covered | Desirable direction | Actual direction during crisis* | | | | | |
|---|---|---|---|---|---|---|---|---|---|---|
| | | | | | Bangladesh | Kyrgyz Republic | Lao | Pakistan | Philippines | Sri Lanka |
| Coping strategies | Livelihood coping strategy (LCS):<br>- None (N)<br>- Stress (S)<br>- Crisis (C)<br>- Emergency (E) | % population | 2022-2023 | ↑ N<br>↓ S,C,E | | N ↓ C ↘<br>S ↑ E → | | N → C ↑<br>S ↗ E ↘ | | |
| | Reduced Coping Strategy Index (rCSI):<br>- Low (L)<br>- Medium (M)<br>- High (H) | % population | 2022-2023 | ↑ L<br>↓ M, H | | ↑ | | → L<br>↑ M<br>↘ H | | |
| Food price volatility | Food price annual inflation | % | 2010-2022 | ↓ | ↗ | ↑ | ↑ | ↑ | ↑ | ↑ |
| | Food Price Anomalies (IFPA), Rice | Index | 2015-2022 | ↓ | ↑ | ↑ | ↑ | ↑ | → | ↑ |

| | | | | | | | | | | |
|---|---|---|---|---|---|---|---|---|---|---|
| Food supply variability | Food supply variability | Kcal/capita/day | 2010-2021 | 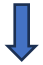 | 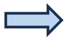 | 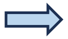 | 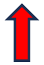 | 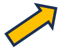 | 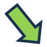 | 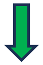 |
| Food security | % population experiencing moderate or severe food insecurity | % population | 2015-2021 | 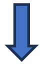 | 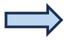 | 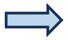 | 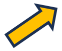 | 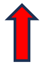 | 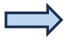 | 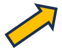 |
| | % population who cannot afford a healthy diet | % population | 2017-2021 | 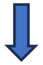 | 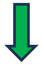 | 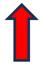 | 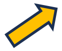 | 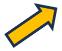 | 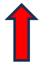 | 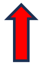 |

* Average crisis period compared to pre-crisis average (2018-2019 depending on data availability)
Desirable direction: denotes a higher value is more desirable,  denotes a lower value is more desirable.
Actual direction : a blue arrow denotes no substantial changes and stable value, a green arrow (up/down) denotes a direction similar to the desirable one, a light green arrow (diagonal up/down) denotes a direction similar to the desirable one but less pronounced, a red arrow (up/down) denotes an opposite direction to the desirable one, an orange arrow (diagonal up/down) denotes an opposite direction to the desirable one but less pronounce

## A.3 – Ranking resilience capacities and agro-food diversity and long-term resilience outcomes

The table presented below illustrates the ranking system applied to assess which countries showed higher or lower resilience capacities and agro-food diversity, as well as long-term resilience outcomes. The following steps were undertaken:

1. A rank ranging from 1 to 6, with 1 denoting the least favorable rank and 6 representing the most favorable rank, was assigned to each value corresponding to the pre-crisis year 2019 for both resilience capacities and agro-food diversity indicators. This ranking was also applied to each value from the most recent available "crisis period" data (from 2020 onwards) for long-term outcomes indicators.
2. In cases where two or more countries exhibited identical values for a specific indicator, an equivalent rank was assigned to each.
3. Within each domain, the total score for each country was computed by summing the ranks assigned to each indicator.
4. The overall score for each country was subsequently determined by dividing the total score by the maximum possible score for that country. The maximum score was set at 36 for both resilience capacities and agro-food diversity and long-term resilience. In cases where a country had missing values for a particular indicator, the maximum score was adjusted to 30.

| | | | Kyrgyz Republic | Philippines | Bangladesh | Lao PDR | Sri Lanka | Pakistan |
|---|---|---|---|---|---|---|---|---|
| **Resilience capacities and agro-food diversity** | | | | | | | | |
| Food produced domestically | Crop production index (2014-2016=100) | Rank 1-6 (6=highest production) | 4 | 3 | 4 | 1 | 2 | 3 |
| | Fertilizer consumption | Rank 1-6 (6=highest consumption) | 1 | 3 | 5 | N/a | 4 | 2 |
| | Livestock production index (2014-2016=100) | Rank 1-6 (6=highest production) | 3 | 1 | 2 | 6 | 4 | 5 |
| Imported food | Food import (volume) NCD-protect | Rank 1-6 for NCD protect only (6=lowest import) | 3 | 4 | N/a | 2 | 5 | 6 |
| Countries' infrastructure level | Mobile cellular subscription | Rank 1-6 (6=highest subscription) | 5 | 6 | 3 | 1 | 4 | 2 |
| Social capital | Social capital index | Rank 1-6 (6=highest social capital) | 5 | 6 | 3 | 1 | 4 | 2 |
| | | **Total Score** | **21** | **23** | **17** | **11** | **23** | **20** |
| | | **Denominator** | 36 | 36 | 30 | 30 | 36 | 36 |
| **Long-term resilience outcomes** | | | | | | | | |
| Food price volatility | Food price inflation | Rank 1-6 (6=lowest inflation) | 4 | 6 | 5 | 3 | 1 | 2 |
| | Food Price Anomalies (IFPA), by type of product (Rice) | Rank 1-6 (6=lowest food price anomaly) | N/a | 4 | 4 | 1 | 2 | 3 |
| | Food Price Anomalies (IFPA), by type of product (Wheat) | Rank 1-6 (6=lowest food price anomaly) | 4 | N/a | 1 | N/a | 2 | 3 |
| Food supply variability | Food supply variability | Rank 1-6 (6=lowest variability) | 2 | 3 | 1 | 4 | 6 | 5 |

| | | | | | | | | |
|---|---|---|---|---|---|---|---|---|
| Food security | % population experiencing moderate or severe food insecurity | Rank 1-6 (6=lowest % ppn food insecure) | 6 | 1 | 4 | 3 | 5 | 2 |
| | % population who cannot afford a healthy diet | Rank 1-6 (6=lowest %ppn who cannot afford healthy diet) | 4 | 2 | 3 | 2 | 5 | 1 |
| | | **Total Score** | **20** | **16** | **18** | **13** | **21** | **16** |
| | | **Denominator** | 30 | 30 | 36 | 30 | 36 | 36 |

# Appendix B: Additional Figures

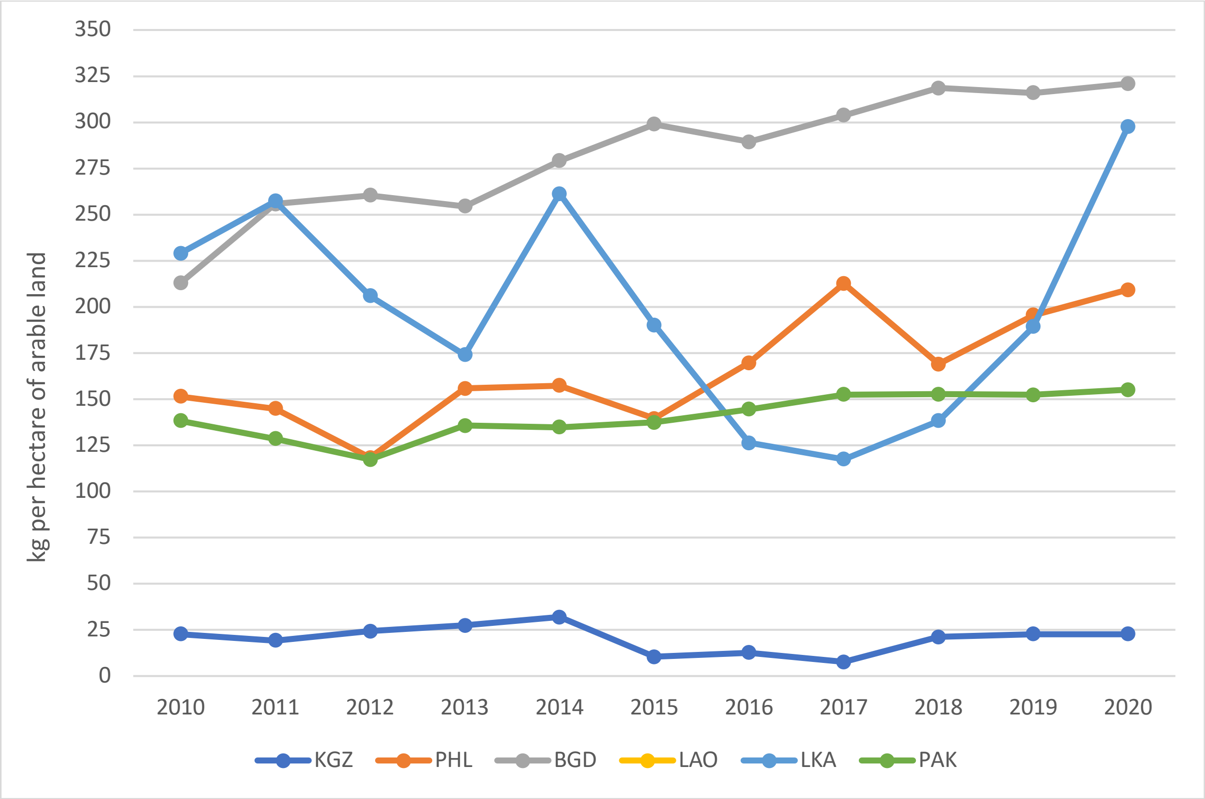


**Figure A. Fertilizer Consumption**

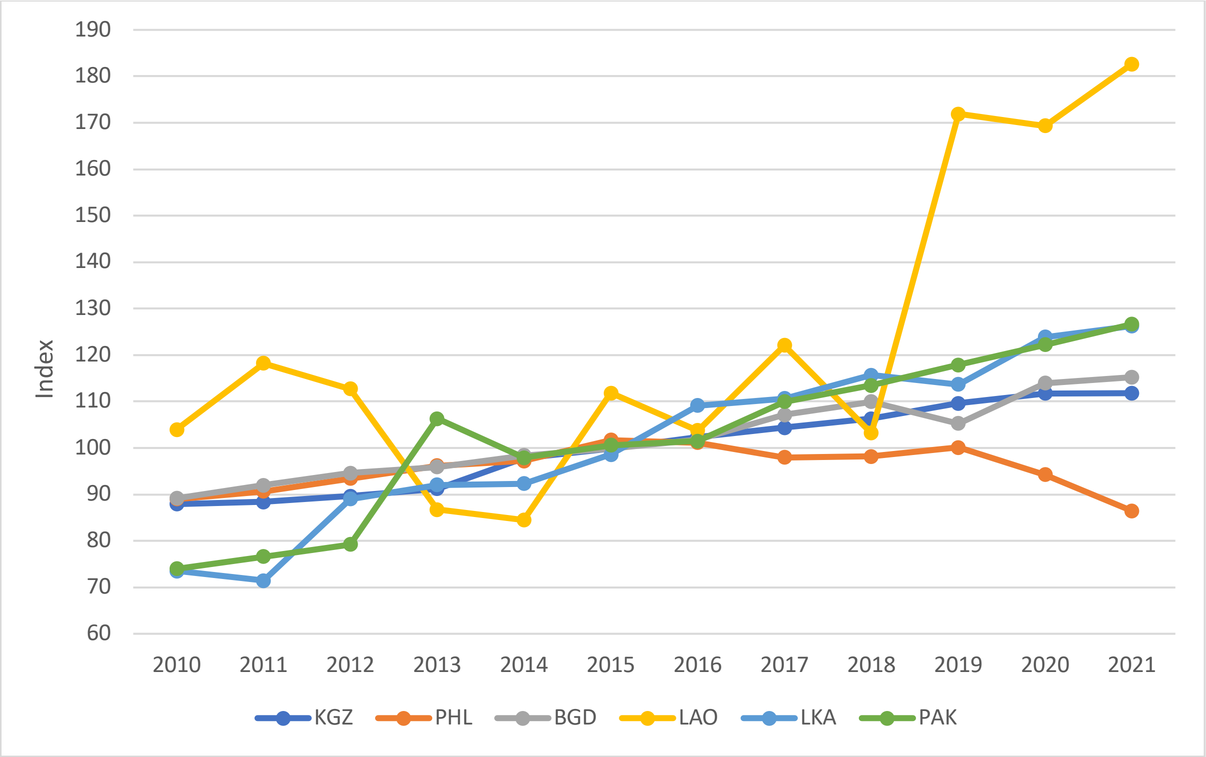


**Figure B. Livestock production Index (2014-2016=100)**

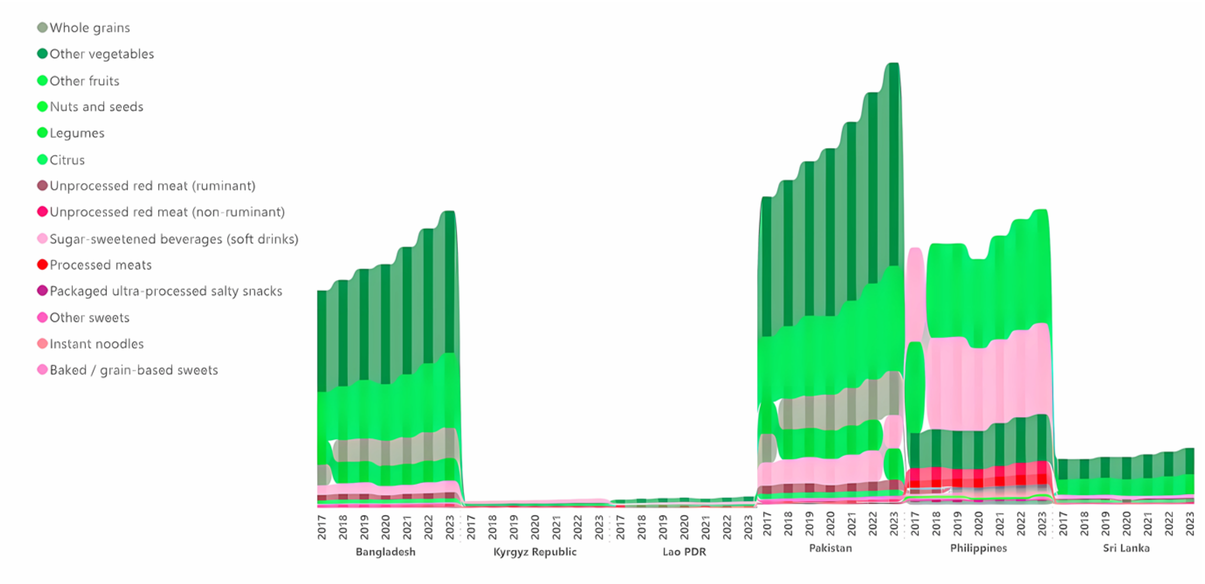


**Figure C. Volume (kg or liters) of NCD-risk and –protect food and beverage sales (2017-2023)**

NCD-Protect: foods protective against noncommunicable diseases (whole grains; legumes/pulses; vitamin A-rich orange vegetables; dark green leafy vegetables; other vegetables; vitamin A-rich fruits; citrus; other fruits; nuts and seeds). NCD-Risk: foods related to noncommunicable diseases (baked/grain-based sweets; other sweets; processed meat; unprocessed red meat - ruminant; unprocessed red -non ruminant; packaged ultra-processed salty snacks; instant noodles; sugar-sweetened beverages).

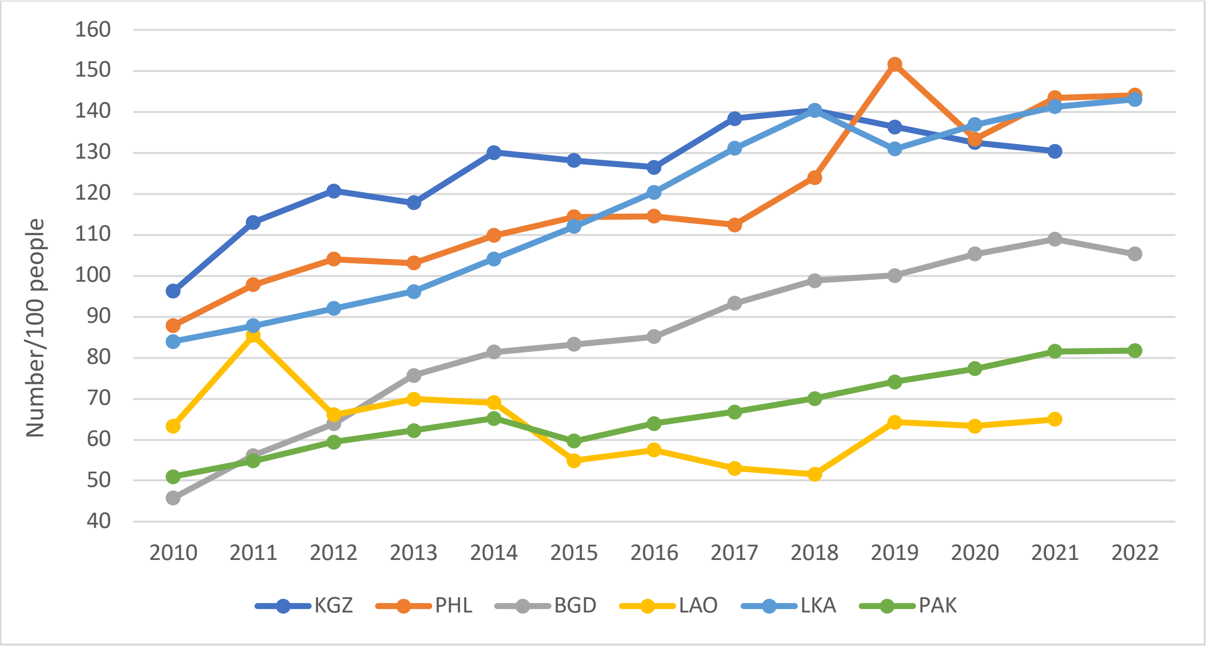


**Figure D. Mobile Cellular Subscription**

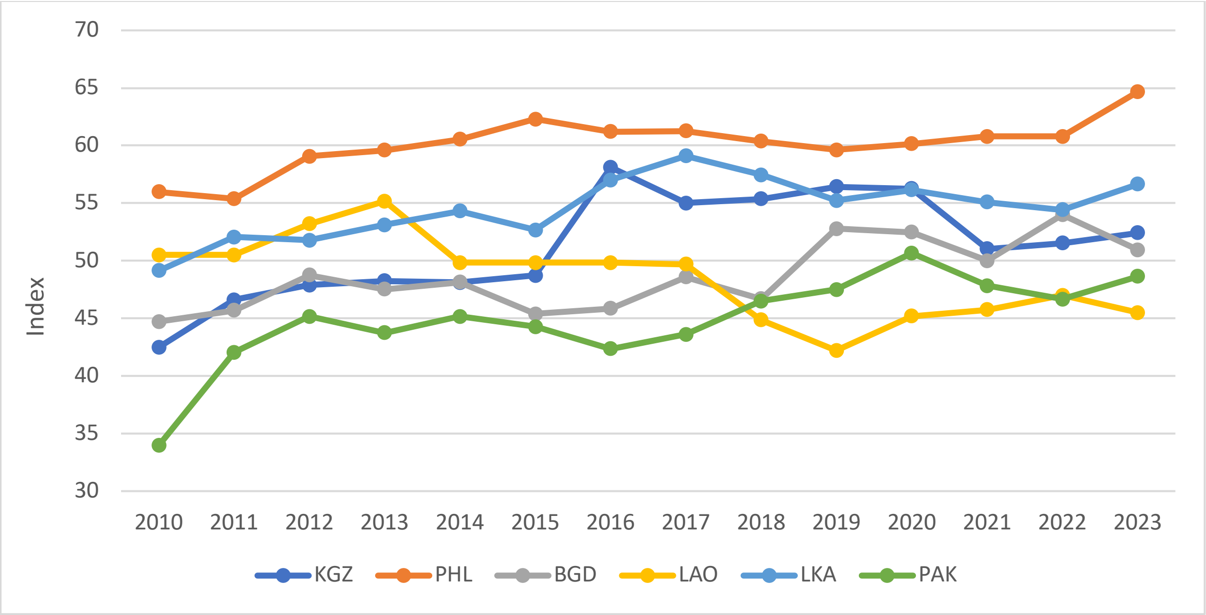


**Figure E. Social Capital Index**

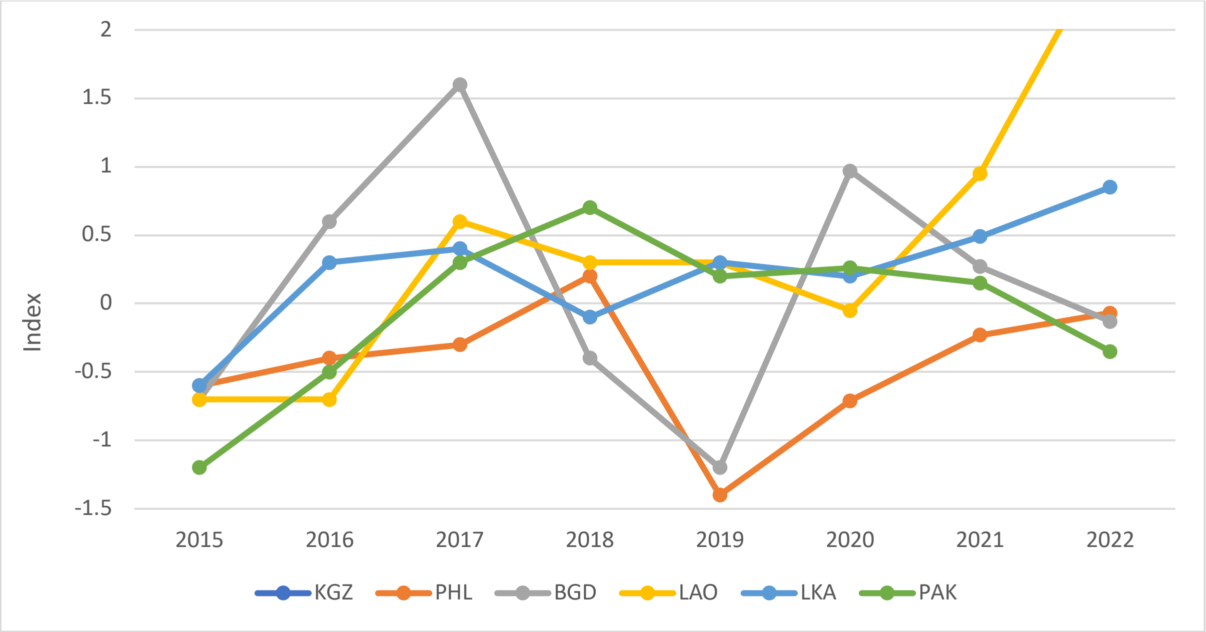


**Figure F. Food Price Anomalies (IFPA), by type of product (Rice)**

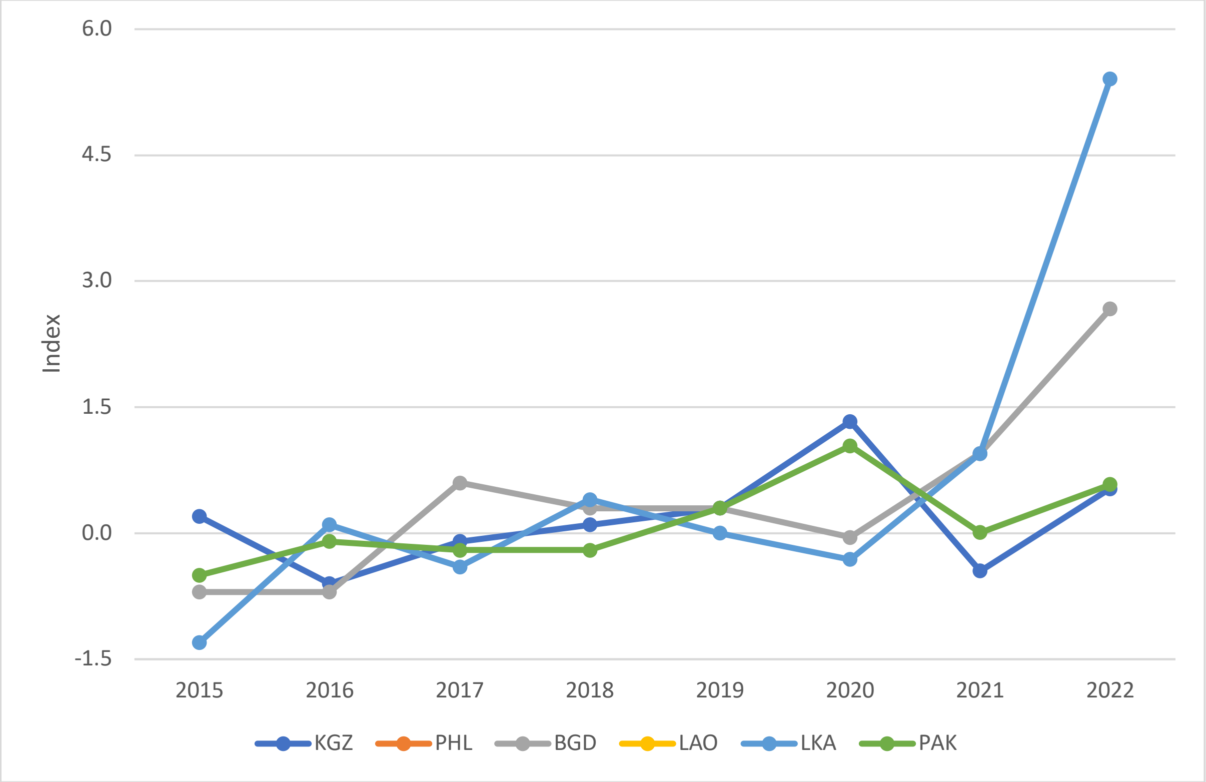


**Figure G. Food Price Anomalies (IFPA), by type of product (Wheat)**

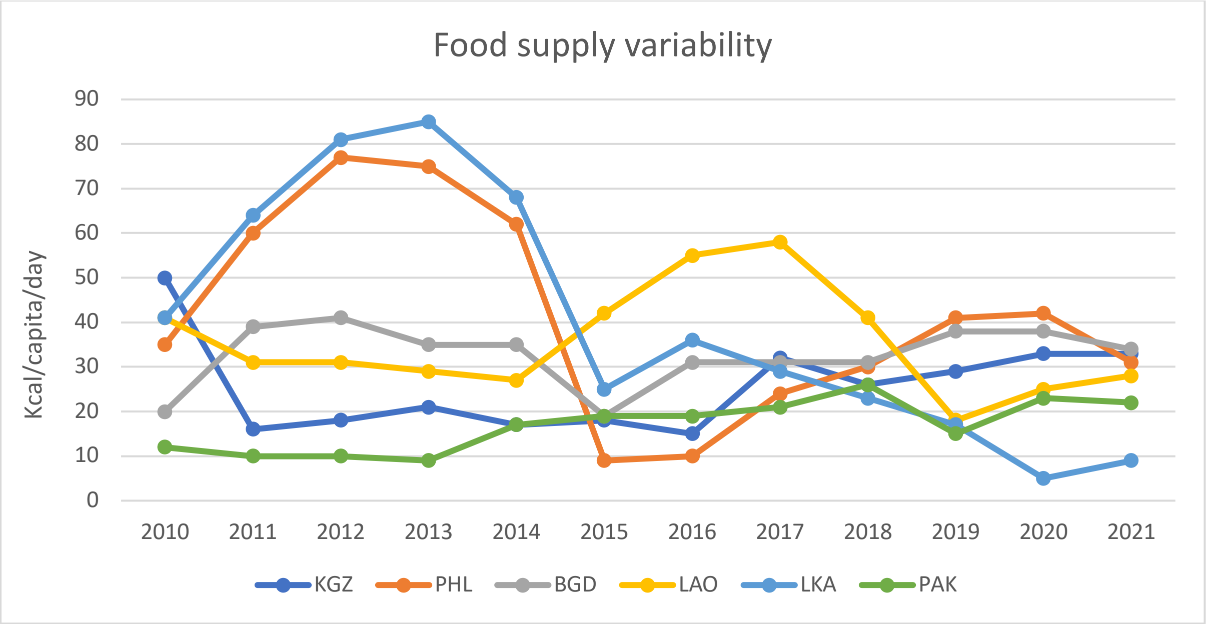


**Figure H. Food supply variability**

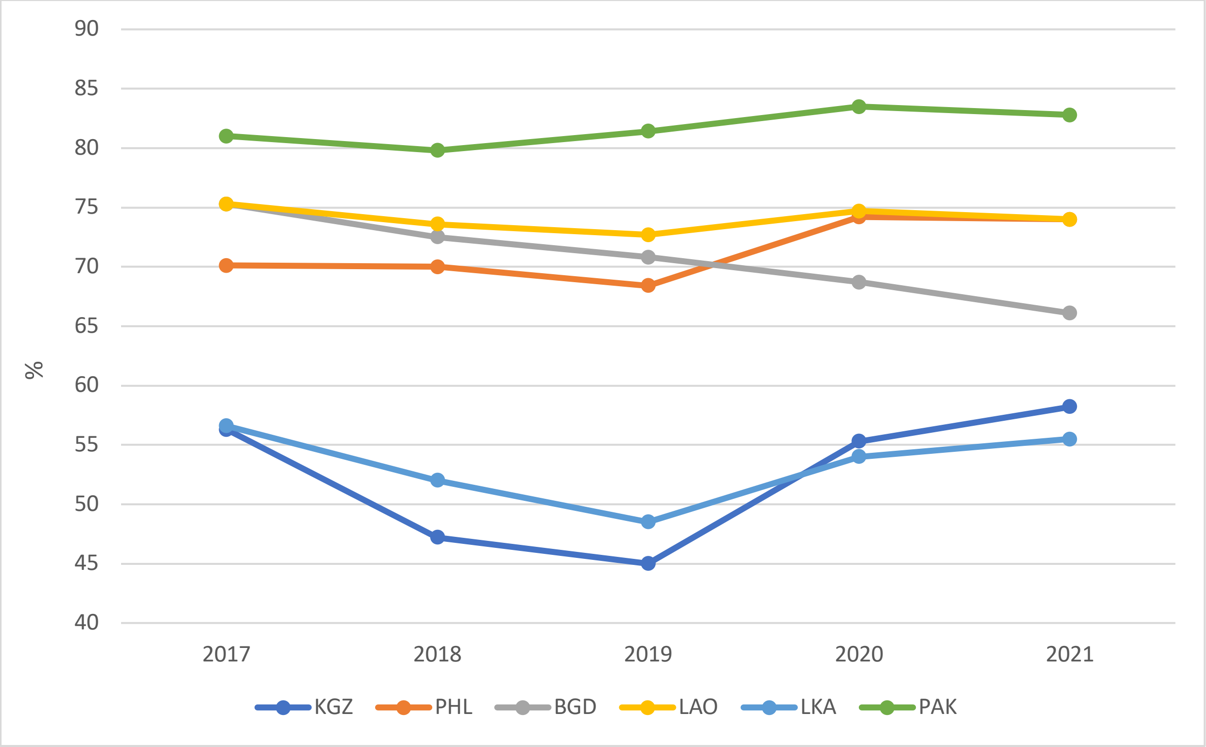


**Figure I. Population who cannot afford a healthy diet (2017-2021)**

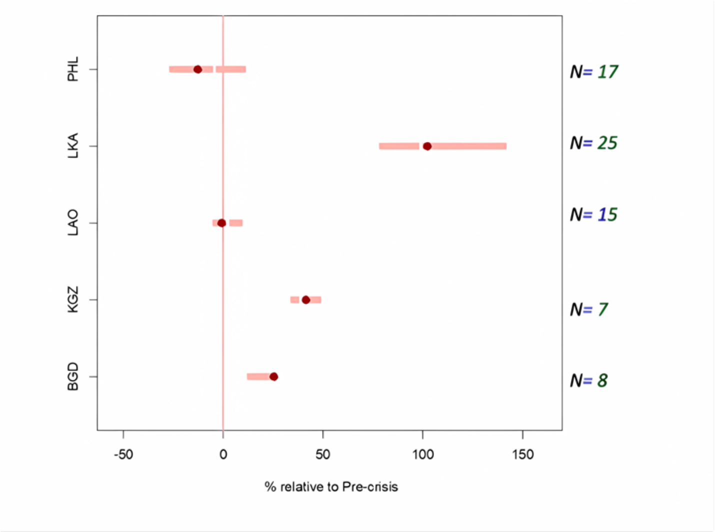


**Figure J. Percent change in Cost of the Diet Energy (2022), relative to most recent pre-crisis data, in Bangladesh, Kyrgyz Republic, Lao PDR, Sri Lanka, Philippines.**

Pre-crisis data: 2015 for Philippines; 2016 for Bangladesh and Sri Lanka; 2017 for Kyrgyz Republic and Lao PDR. N is the number of sub-national values. Dots in red are the national level values.

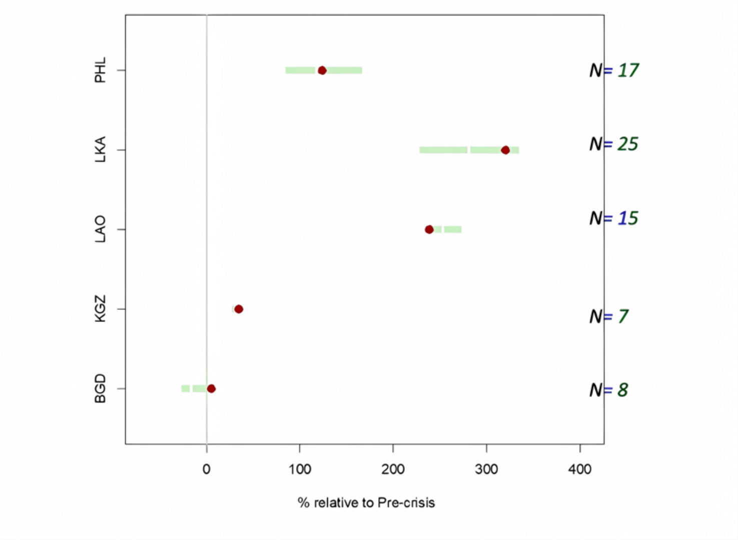


**Figure K. Percent change in Cost of the Nutritious Diet (2022), relative to most recent pre-crisis data, in Bangladesh, Kyrgyz Republic, Lao PDR, Sri Lanka, Philippines.**

Pre-crisis data: 2015 for Philippines; 2016 for Bangladesh and Sri Lanka; 2017 for Kyrgyz Republic and Lao PDR.
N is the number of sub-national values. Dots in red are the national level values.
